# Adipose tissue-derived microRNAs as epigenetic modulators of type 2 diabetes

**DOI:** 10.1186/s12916-025-04560-7

**Published:** 2025-12-09

**Authors:** Ratika Sehgal, Neele Haacke, Alice Maguolo, Fiorella A. Solari, Markus Jähnert, Pascal Gottmann, Emma Nilsson, Allan Vaag, Pamela Fischer-Posovszky, Anja Werberger, Andreas L. Birkenfeld, Andreas Fritsche, Hans-Ulrich Häring, Albert Sickmann, Heike Vogel, Charlotte Ling, Meriem Ouni, Annette Schürmann

**Affiliations:** 1 Department of Experimental Diabetology, German Institute of Human Nutrition Potsdam- Rehbruecke, Nuthetal, Germany; 2 German Center for Diabetes Research (DZD), Munich-Neuherberg, Germany; 3 Division of Endocrinology and Diabetology, Department of Internal Medicine 1, University Hospital Ulm, Ulm, Germany; 4 Epigenetics and Diabetes Unit, Department of Clinical Sciences, Lund University Diabetes Centre, Scania University Hospital, Lund University, Malmö, Sweden; 5 Leibniz Institut Für Analytische Wissenschaften-ISAS-E.V., Dortmund, Germany; 6 Department of Clinical Sciences in Malmö, Lund University Diabetes Centre, Scania University Hospital, Malmö, Sweden; 7 Copenhagen University Hospital - Steno Diabetes Center Copenhagen, Herlev, Denmark; 8 Department of Pediatrics and Adolescent Medicine, Ulm University Medical Center, Ulm, Germany; 9 German Center for Child and Adolescent Health (DZKJ), Partner Site Ulm, Ulm, Germany; 10 Institute of Diabetes Research and Metabolic Diseases (IDM) of the Helmholtz Center Munich, University of Tübingen, Tübingen, Germany; 11 Department of Internal Medicine IV, University Hospital Tübingen, Tübingen, Germany; 12 Medizinische Fakultät, Medizinische Proteom-Center, Ruhr-Universität Bochum, Bochum, Germany; 13 Research Group Nutrigenomics of Obesity, German Institute of Human Nutrition Potsdam-Rehbruecke, Nuthetal, Germany; 14 Research Group, Epigenetic of Obesity and Diabetes, German Institute of Human Nutrition Potsdam-Rehbruecke, Nuthetal, Germany; 15 Institute of Nutritional Science, University of Potsdam, Nuthetal, Germany

**Keywords:** Adipose tissue, MicroRNA (miRNA), Type 2 diabetes (T2D), Imprinting, Epigenetics, Multiomics, New Zealand Obese (NZO) mice, Discordant monozygotic twins

## Abstract

**Background:**

White adipose tissue (WAT) dysfunction including an aberrant expression of miRNAs is strongly associated with the risk of developing type 2 diabetes (T2D), with limited evidence linking early changes in the WAT-derived miRNAs and T2D. The present study aims to identify early miRNome changes prognostic for T2D in mice and humans.

**Methods:**

Gonadal (g) WAT of diabetes-resistant and diabetes-prone mice were subjected to multi-omics analyses (transcriptome, miRNome, methylome, proteome). Metabolic phenotypes linked with T2D were correlated with adipose tissue miRNA expression and DNA methylation from 14 monozygotic twin pairs discordant for T2D. Plasma miRNA levels from females at high risk of developing T2D (TÜF study) were included.

**Results:**

Adipose tissue of the diabetes-susceptible mice was less insulin sensitive with ~ 200 differentially expressed mature miRNAs compared to diabetes-resistant mice. Integrative analysis of miRNome-transcriptome-proteome identified 227 proteins involved in amino acid metabolism, inflammation, signalling pathways, and insulin resistance. More than 20 differentially expressed miRNAs are located in the imprinted region *Dlk1-Gtl2* and *Mest* (miR-335) potentially regulated by DNA methylation. Imprinted miRNAs also exhibited similar alterations in adipose tissue from monozygotic twin pairs discordant for T2D, with miR-335 expression altered only in females. Moreover, plasma levels of miR-335-5p were negatively correlated with fasting blood glucose in females at high risk of developing T2D.

**Conclusions:**

Early alterations of WAT-derived miRNAs such as miR-335-5p could contribute to systemic metabolic changes associated with the risk of developing T2D.

**Supplementary Information:**

The online version contains supplementary material available at 10.1186/s12916-025-04560-7.

## Background

The existing global healthcare system is overwhelmed by a profound rise in the prevalence of type 2 diabetes (T2D), accounting for one death every 5 s and imposing a global financial burden [1]. These alarming trends demand the development of novel treatment strategies and early diagnostic markers for T2D in addition to reinforcing a healthier lifestyle [2]. Impaired adipocyte plasticity and adipose tissue inflammation are key hallmarks mediating the course of metabolic derangements in obesity, leading to insulin resistance and T2D [3]. In this context, over the past years, the therapeutic potential of several microRNAs (miRNAs) has been investigated [4]. MiRNAs are ⁓22 nucleotides long non-coding RNAs that target and modify the expression of multiple genes and proteins [5].

For instance, the let-7 family of miRNAs has been recognized as a potent regulator of insulin signalling and glucose metabolism by targeting the transcription factor HMGA2 (high mobility group AT-hook 2) amongst others [6]. Specific miRNAs such as miR-103/107 have been identified to trigger endoplasmic reticulum stress and apoptosis of preadipocytes [7]. Adipose tissue dysfunction in obesity was associated with elevated miR-30/125a-5p, both known to trigger inflammation and insulin resistance [8]. Adiponectin production has been found to be heavily regulated by miRNAs such as miR-193b [9]. There is also ample evidence to support the idea that adipose tissue remodelling is epigenetically driven, resulting in a heterogeneous presentation of obesity and its related diseases [10–12]. Not all individuals with obesity develop T2D, and not all individuals with T2D are obese, and these differences are partially mediated by epigenetic mechanisms [13, 14]. It is therefore necessary to identify early epigenetic modulators of these metabolic adaptations. Moreover, further research is required to understand and establish the biological significance of adipose tissue-derived miRNAs in T2D and their diagnostic and therapeutic potential.

It is also well known that adipose tissue is a major contributor to a circulating miRNA pool, and the adipose tissue miRNA profile is severely altered in case of T2D and obesity, mainly in response to calorie excess [15, 16]. More recently, researchers have also identified that adipose tissue secretome regulates several physiological processes, including glucose and lipid metabolism, insulin sensitivity, inflammation, and energy homeostasis [17]. Although several studies investigated the association between circulating miRNAs and T2D risk, rarely has the source of detected miRNAs been explored, and thus it remains a challenging question. Additionally, studies linking circulating miRNA profile and prediabetes are limited. As adipose tissue is one of the most dynamic tissues in which many molecular changes occur before the development of T2D, investigation of its miRNAs may reveal potential early and stable miRNAs that can serve as biomarkers for T2D.

In the present study, we investigated gonadal white adipose tissue (gWAT)-derived miRNAs as epigenetic modulators of T2D using genetically identical New Zealand Obese (NZO) female mice differing in their susceptibility to develop T2D. On a 60% high-fat diet, about 40% of the mice develop T2D, whereas the remaining mice are protected from hyperglycemia; in contrast, nearly all male NZO develop T2D under these conditions. We are able to distinguish between diabetes-resistant and -prone female mice already at week 10, before the onset of T2D [18, 19]. Thus, this mouse model offers a unique opportunity to study epigenetic mechanisms before the onset of the disease affecting cellular and molecular adaptations in metabolically active tissues like adipose tissue [18, 19]. Conserved and functionally relevant miRNAs were further screened for changes in adipose tissue of monozygotic twin pairs discordant for T2D from Scandinavian Twins registries to translate current research findings to humans [20]. In addition, the status of selected miRNAs in plasma of females at high risk of developing T2D from the Tübingen lifestyle intervention (TÜF) study was investigated.

## Methods

### Animal study design

The animal study protocol and experimental design have been detailed in previous publications [18, 19, 21]. After 5 weeks of high-fat diet (HFD) feeding, female New Zealand Obese (NZO) mice were phenotypically classified as either diabetes-prone (DP) or diabetes-resistant (DR) based on a risk score [19]. For the majority of the life span, the DR group remains normoglycemic, while DP mice develop hyperglycemia later in life. For the current study, the gWAT was harvested and plasma samples were collected at week 10. All procedures involving animals were approved by the ethics committee of the State Agency of Environment, Health and Consumer Protection (2347–28–2014, State of Brandenburg, Germany).

### Adipose tissue insulin resistance index & adipocyte size measurement

The quantitative determination of plasma insulin was performed with Mouse Ultrasensitive Insulin ELISA by ALPCO (catalog number: 80-INSMUE01; USA) from 15 DR and 13 DP mice, as described earlier [18]. Plasma free fatty acid levels were measured using the NEFA-HR assay (reagent 1: 434–91,795; reagent 2: 436–91,995; standard: 270–77000; Neuss, Germany), as described previously [19]. The adipo-IR index was calculated by multiplying the plasma insulin levels with the free fatty acid measurements. Adipose tissue was fixed in 4% formaldehyde for 24 h at room temperature, followed by dehydration, paraffin embedding, sectioning, and staining (cytosol-eosin and nuclei-hematoxylin). The images were captured using a light-optical microscope Eclipse E800 (Nikon, Chiyoda, Japan), and adipocyte size was measured with ImageJ (National Institutes of Health, Bethesda, USA).

### Genome-wide RNA, small-RNA and DNA methylation profiling of gWAT

Total RNA was extracted from the gWAT (*n* = 15/group) using a miRNeasy kit (Qiagen, Germany) as per the manufacturer’s instructions and quantified as detailed previously [21]. RNA samples were sequenced by BGI (Shenzen, China) using BGISEQ for whole-genome transcriptome analysis and small RNA sequencing using BGISEQ-500 (DNBSEQ-G50) Sequencing V.1 technology. Differential expression analysis for transcripts was done using DESeq2v1.34.0 in R (v.4.1.2); minimum mean FPKM > 1; Wald test and Benjamini–Hochberg method to adjust for multiple testing and differential miRNA expression analysis was done using EdgeR v3.36.0 [22]. The criteria for the minimum read count was set to mean read count > 10 and 29 out of 30 analysed samples were included for the statistical analysis. The detailed methodology can be found in the previous publication [21]. Of note, the small RNA sequencing data were annotated for both mature and hairpin miRNAs based on the miRDeep2 pipeline, as described elsewhere [23].

Secondly, genomic (g) DNA was isolated from gWAT (*n* = 6/group) using the ReliaPrep™ gDNA Tissue Miniprep System (Promega). The samples were sent to LIFE & BRAIN GENOMICS GmbH (Bonn, Germany) for genome-wide analysis using the Illumina Methylation Array (MM285, Illumina, San Diego, CA, USA). In brief, a specific amount of gDNA was bisulfite converted, amplified, fragmented, hybridized onto the array chip, and scanned as described earlier [21]. Differentially methylated CpG sites were identified using a mixed linear model with the limma R package.

### Weighted miRNA correlation network and transcription factor binding motifs enrichment analyses

In line with previous studies, Weighted Gene Co-expression Network Analysis (WGCNA) was employed to analyze our miRNome data (WMCNA) [24] (R version 4.3.1; WGCNA version 1.71). Throughout the analysis, the guidelines established by Horvath et al. were adhered to [25]. Optimization for scale-free topology yielded an optimal soft-thresholding power (β) of 12. In addition to default parameters, we utilized a signed network and set the minimum module size to ten. Hub miRNAs were defined by their top 5 ranking in terms of connectivity and a module membership (MM) value > 0.75. Visual representations were generated using the R package igraph (version 1.5.1). Furthermore, to evaluate if miRNAs that cluster on specific chromosomes are also regulated by a common transcription factor (TF), we screened for putative TF binding motifs 500 bp upstream of the transcription start site (TSS) of the miRNA. The TF binding motifs were retrieved from JASPAR 2024 as position weight, and binding sites were predicted via TFBSTools (version 1.40). To assess whether a miRNA promoter was enriched for a specific binding motif, we compared the number of motifs in that promoter with the number of motifs across promoters of all other miRNAs. This comparison was conducted using the Crawford-Garthwaite test, with a significance threshold set at 10^–6^ using R-package psycho (version 0.6.1). For convenience, TF names were set as capital letters even if binding motifs were originally received from mouse experiments.

### Proteomics analysis

For label-free proteome analysis, frozen powdered gWAT tissue samples were lysed, followed by protein concentration measurement using the bicinchoninic acid assay (Pierce, Thermo Fisher Scientific, Rockford, USA). Thereafter, cysteines were reduced, and free sulfhydryl groups were alkylated, and samples (55 µg) were processed using the S-trap Mini Column Digestion Protocol (PROTIFI, Farmingdale, NY, USA) according to the manufacturer’s instructions, with slight modifications as detailed in Additional file 1: Methods [26–28]. Digestion quality control was performed via a monolithic column–HPLC [27]. NanoLC–MS/MS analysis was done using a U3000 RSLCnano online-coupled (Thermo Scientific, Bremen, Germany) to TIMS-TOF mass spectrometer (Bruker Daltonics, Bremen, Germany), with further details elaborated in Additional file 1: Methods. Raw data were analysed by Spectronaut Pulsar (Biognosys), using a mouse database (swissPROT_tremBl) following specific parameters. Results files were exported, based on PG protein quantification fold change (FC), and *p*-values (*p*) were calculated using an unpaired *t*-test for DP versus DR samples. In total, 6032 proteins were quantified, from which 1606 were up or down-regulated with *p* < 0.05, and 407 with |log_FC_ (DP/DR)|> 0.5 and *p* < 0.05. Raw data and search results are deposited in the ProteomeXchange repository (project accession number PXD057291).

### miRCURY assay-based validation for selected miRNAs

The miCURY LNA RT Kit (Qiagen, Cat. No. 339340, Hilden, Germany) was used to synthesize cDNA, and the miCURY LNA Sybr Green PCR Kit (Qiagen, Cat. No. 339346, Hilden, Germany) was used for the qPCR. For each primer assay and sample, duplicates were made, and RNU6-1/U6 (RNA, U6 Small Nuclear 1) served as the endogenous control. The following primer assays were used (all fully conserved in humans except miR-342): hsa-miR-100-5p, hsa-miR-130a-5p, hsa-miR-142-5p, hsa-miR-146a-5p, has-miR-146b-5p, hsa-miR-196b-5p, hsa-miR-199a-5p, hsa-miR-30b-5p, hsa-miR-335-5p, has-miR-376a-3p, hsa-miR-409-3p, hsa-miR-455-5p, hsa-miR-let-7i-5p, and mmu-miR-342-5p (Additional file 1: Methods).

### miRNA-mRNA-protein target prediction and pathway enrichment analysis

The miRNA-mRNA target prediction was performed to identify putative yet experimentally validated targets for miRNAs based on a workflow described previously [18]. Precisely, one mRNA is considered a putative target based on its prediction in at least three out of five queried prediction tools and experimental validation evidence based on TarBase.v9. The mRNAs that were also found to be differentially expressed in the current dataset were then subjected to pathway and gene ontology enrichment analysis using the DAVID tool. An additional layer of adipose tissue proteomics data was overlaid onto the predicted miRNA-mRNA targets using the R package igraph version 2.0.3 to get a holistic molecular snapshot of early prediabetic adipose tissue in NZO female mice.

### Human cohorts

#### Monozygotic twin pairs discordant for T2D

Fourteen monozygotic twin pairs discordant for T2D were included in the present analysis from our previously published study [20, 29]. Total RNA was extracted from frozen subcutaneous adipose tissue biopsies from 12 of the 14 discordant twin pairs using the miRNeasy kit (Qiagen, Hilden, Germany), and miRNA expression was analysed by miRNA 3.0 arrays (Affymetrix, Santa Clara, CA) as detailed elsewhere [20]. In addition, genomic DNA was extracted from 14 discordant twin pairs with the DNeasy Blood and Tissue kit (Qiagen) and subjected to DNA methylation analysis using Infinium HumanMethylation450 BeadChips (Illumina). The detailed bioinformatics workflow and the clinical characteristics of the cohort, as well as some of the miRNA and DNA methylation data, have been published previously [20, 29]. To identify the differences between the discordant twins, a paired two-tailed Wilcoxon test was performed, and Spearman statistics were used for the correlation analyses with blood glucose and HbA1c levels. We examined correlations between epigenetic markers and T2D-related phenotypes (like glucose and HbA1c levels) both dependently and independently of the twin pairs, with the aim of highlighting epigenetic differences due to environmental factors, lifestyle, or disease status that are not necessarily paired in the same way as genetic data. Analyses were performed with SPSS (IBM) version 29.

#### German cross-sectional TÜbingen Family study for type 2 diabetes (TÜF)

A subgroup comprising 99 females at high risk of developing T2D was included from the TÜF cohort comprising more than 3000 non-related individuals [30]. The plasma miRNA expression profile was correlated (Spearman statistics) with the fasting blood glucose levels in age- and BMI-matched individuals. All the above-mentioned study protocols were approved by the relevant local medical ethics committee. Written informed consent was obtained from all the study participants, and the study was carried out in accordance with the Declaration of Helsinki.

### Effect of miR-335 mimic transfection in 3T3-L1 cells

The 3T3-L1 fibroblasts (ATTC® CL-173™) were cultured in standard conditions and were seeded on collagen-coated (Collagen A, Biochrom GmbH, Berlin, Germany) plates and grown until sub-confluent, as detailed earlier [21]. Using Lipofectamine 2000 (Invitrogen) transfection reagent, 20 nM miRNA mimic miR-335-5p (YM00473600-ADA, Qiagen) was transfected into these 3T3-L1 fibroblasts for 48 h. The scrambled miRNA (YM00479902-ADA, Qiagen) was used as a control. Differentiation was started using IMDM/FBS containing 200 nM insulin (Roche), 0.5 mM 3-isobutyl-1-methylxanthine (Sigma-Aldrich), 2 mM rosiglitazone (Sigma-Aldrich) and 250 nM dexamethasone (Sigma-Aldrich) and cultured for 3 days. Medium was replaced with IMDM/FBS containing 200 nM insulin every day for 2 days, followed by 1 day in IMDM/FBS and 1% penicillin/streptomycin (P/S). Cells were harvested before induction of differentiation (d0) and during the differentiation at day 1 to day 6 (d1–d6). Changes in gene/miRNA expression were evaluated using qPCR (Additional file 1: Methods) and data were normalized with appropriate endogenous controls. RNA sequencing was conducted at day 3 and day 6 of differentiation. Analysis was performed by BGI using DEseq2 and genes were considered as significant differentially expressed with *q* < 0.01.

To assess insulin sensitivity of 3T3-L1 cells treated with miR-335-5p, adipocytes at day 5 of differentiation were cultured in 250 µL media for 48 h in 24-well plates before being serum-starved in IMDM containing 0.2% BSA for 6 h and then stimulated with 0.5 or 100 nM insulin for 20 min. Cells were washed three times with ice-cold PBS and lysed in RIPA buffer containing protease and phosphatase inhibitors (Thermo Fisher Scientific).

The triglyceride levels of 3T3-L1 adipocytes at day 3 and day 6 of differentiation were analysed using the Randox Triglycerides assay (GPO-PAP, TR210) according to the manufacturer’s protocol, as detailed earlier [21]. For Oil Red O staining at day 3 and day 6 of differentiation, the cells cultured on cover slips were washed with PBS and fixed with 4% paraformaldehyde for 10 min and washed with PBS three times for 5 min. After fixation, cells were washed with water and 60% isopropanol for 5 min. A freshly prepared and filtered Oil Red O working solution (6:4 dilution of stock 75 mg Oil Red O (Sigma-Aldrich) in 15 mL isopropanol and filtered after 24 h) was used and incubated for 10 min at room temperature. Cells were rinsed with 60% isopropanol two times and washed with water. Finally, cells were mounted with aqueous mounting medium (Dako Faramount, S3025) and lipid droplets were visualized using a light microscope.

### Luciferase reporter assay in HeLa cells

HeLa cells were seeded in 96-well plates and grown overnight to ∼70% confluency. Cells were transfected with either the binding sites of *Gna12* (GeneCopoeia provided by BioCat GmbH, Germany) or *Gga3* (Origene Technologies, Inc, USA) plasmids together with the mmu-miR-335-5p mimic or the non-targeting miRNA mimic (YM00473600-ADA, Qiagen) using Lipofectamine 2000 for 48 h. Each condition (*Gna12* + miR-335-5p-mimic; *Gna12* + non-targeting miRNA mimic; *Gga3* + miR-335-5p-mimic; *Gga3* + non-targeting miRNA mimic) was transfected in three to four replicate wells and repeated in three independent experiments. Selected sequences cloned into the luciferase reporter constructs are available upon request.

## Results

### Study design

**Fig. 1:**
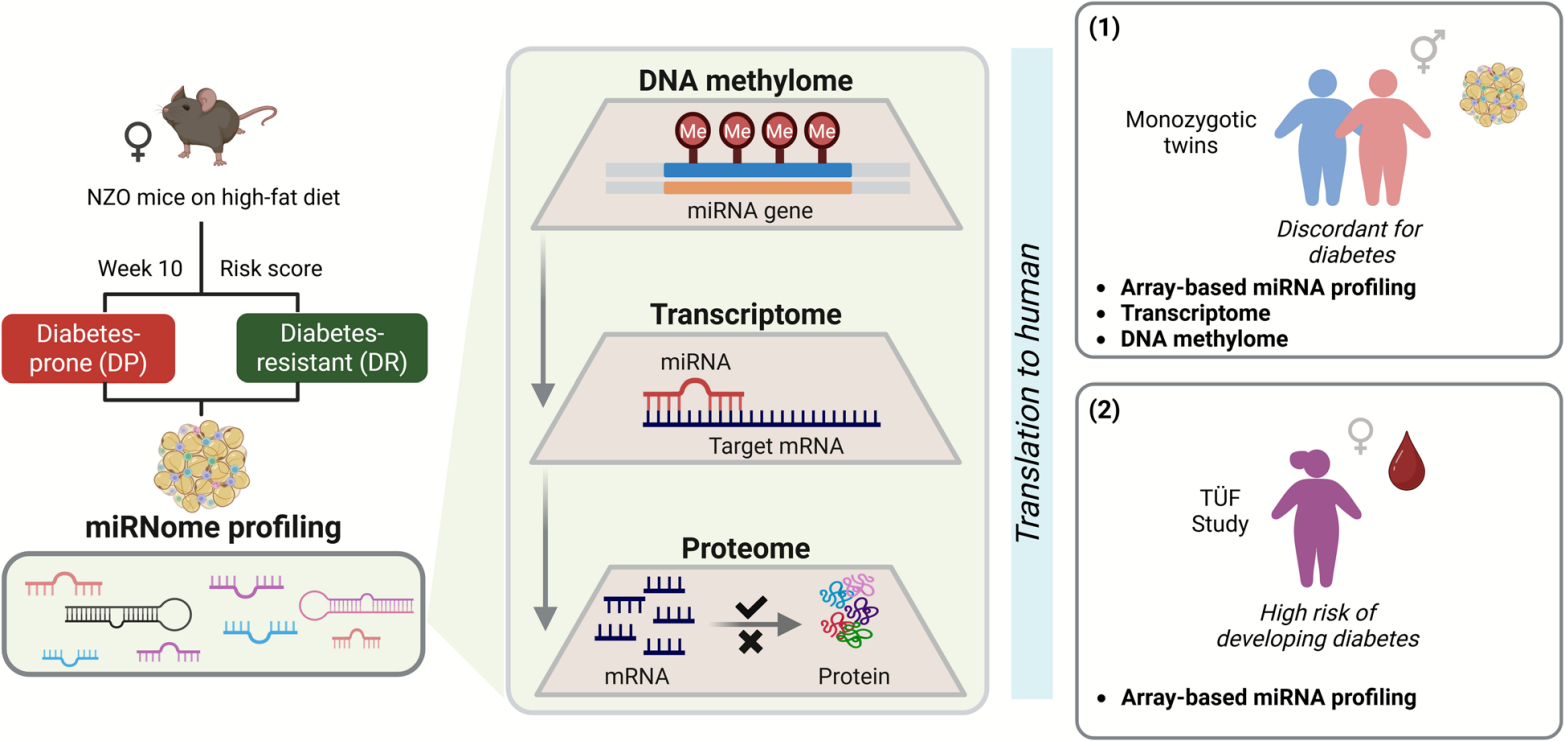
Study design. Total RNA and genomic DNA were isolated from the gonadal white adipose tissue (gWAT) of 10-week-old New Zealand Obese (NZO) female mice phenotypically characterized as either diabetes-resistant (DR) or -prone (DP) based on a risk score. The miRNA expression was analysed using small-RNA sequencing, mRNA expression using RNA sequencing, DNA methylation analysis using the Infinium Mouse Methylation BeadChip array (MM285), and proteomics using LC–MS/MS. The findings from the mouse model were further translated into humans using two independent cohorts: (1) monozygotic twin pairs discordant for T2D from Scandinavian twin registries and (2) TÜF study. Figure created with BioRender.com

To identify epigenetic factors modulating adipose tissue function and the risk of developing T2D, the present study took advantage of genetically identical female New Zealand Obese (NZO) mice differing in their T2D susceptibility [18, 19]. This specific mouse model provides a unique opportunity to study the regulatory epigenetic mechanisms that influence cellular and molecular adaptations in metabolically active tissues, such as adipose tissue, already before the onset of T2D [31]. To this end, gWAT from 15 diabetes-prone (DP) and 15 diabetes-resistant (DR) mice were analysed for changes in the miRNome and transcriptome profiles (Fig. 1). Subsequently, gWAT from some of these mice were subjected to DNA methylation and proteomics analyses. These omics layers were then combined to decipher miRNA-mRNA-protein networks and to identify potential biomarkers for T2D (Fig. 1). The findings from the mouse model were translated to humans using two independent human cohorts: (1) The disease phenotype and contribution of changes in DNA methylation were derived from adipose tissue transcriptome and DNA methylome profiles of monozygotic twin pairs discordant for T2D. (2) To establish the relevance of identified miRNAs as putative biomarkers in the context of T2D, the plasma miRNA abundance was studied in prediabetic females at high risk of developing T2D from the TÜF study (Fig. 1).

### Adipose tissue insulin sensitivity and inflammation define the risk of T2D

**Fig. 2:**
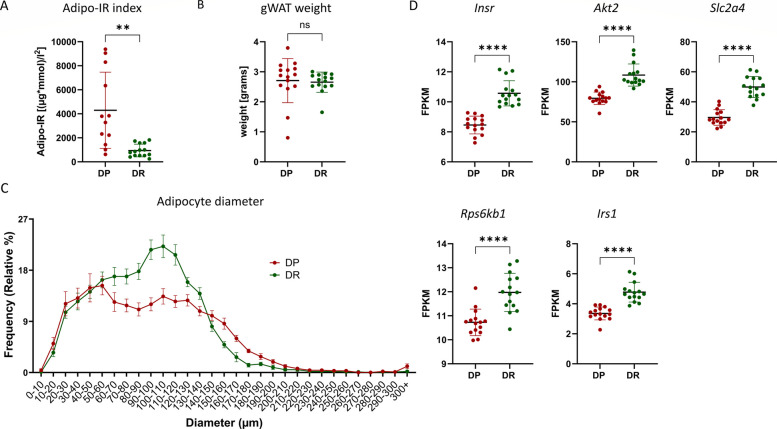
Altered adipose tissue insulin sensitivity before the onset of hyperglycemia in DP mice. **A** Adipose tissue insulin resistance index (Adipo-IR) based on plasma insulin and plasma free fatty acids levels in DP and DR mice. **B** gWAT weight in grams for DP and DR mice harvested at week 10. **C** Adipocyte size distribution is shown as the frequency (relative %, *y*-axis) of the adipocytes belonging to a specific diameter bin (*x*-axis). **D** Expression of indicated genes shown as fragments per kilobase of transcript per million mapped reads (FPKM) detected by RNA sequencing. Data are shown as mean ± SD (**A**,** B**, and **D**) and mean ± SEM (**C**); unpaired *t*-test with Welch’s correction (**A** and **B**); two-way ANOVA with post hoc Tukey test (**C**); DESeq2 Wald test statistics with *p*-value adjusted for multiple comparisons using Benjamini–Hochberg method (**D**); ns, not significant, *p < 0.05, **p < 0.01, ****p < 0.0001

Since the mouse model included in the present study reflects an early prediabetic state at 10 weeks of age, the insulin sensitivity of the adipose tissue was evaluated. The adipose tissue insulin sensitivity markedly differs between the DP and DR mice (Fig. 2A), without any change in the gWAT weight (Fig. 2B). The adipose tissue insulin resistance (Adipo-IR) index was calculated using plasma insulin and free fatty acid levels (Additional file 1: Fig. S1), both of which were found to be significantly higher in DP mice compared to DR. Specifically, the mean Adipo-IR index was ~ 4 times higher in DP mice, providing evidence of pronounced adipose dysfunction and reduced insulin sensitivity in DP compared to DR (Fig. 2A). The adipocyte size measurement revealed a trend (no significant difference) towards a reduced number of smaller adipocytes (diameter < 100 µm) and more larger adipocytes (diameter > 300 µm) in DP gWAT sections compared to DR mice (Fig. 2C). Lastly, these findings were further supported by the altered expression of key insulin-signalling genes in the adipose tissue of DP mice (Fig. 2D). The mRNA expression of *Insr*, *Akt2*, *Slc2a4*, *Rps6kb1*, and *Irs1* genes was found to be lower in gWAT of DP mice compared to DR, overall implying a less insulin-sensitive adipose tissue in DP mice compared to DR.

### Adipose tissue miRNA profile is altered before T2D manifestation

**Fig. 3:**
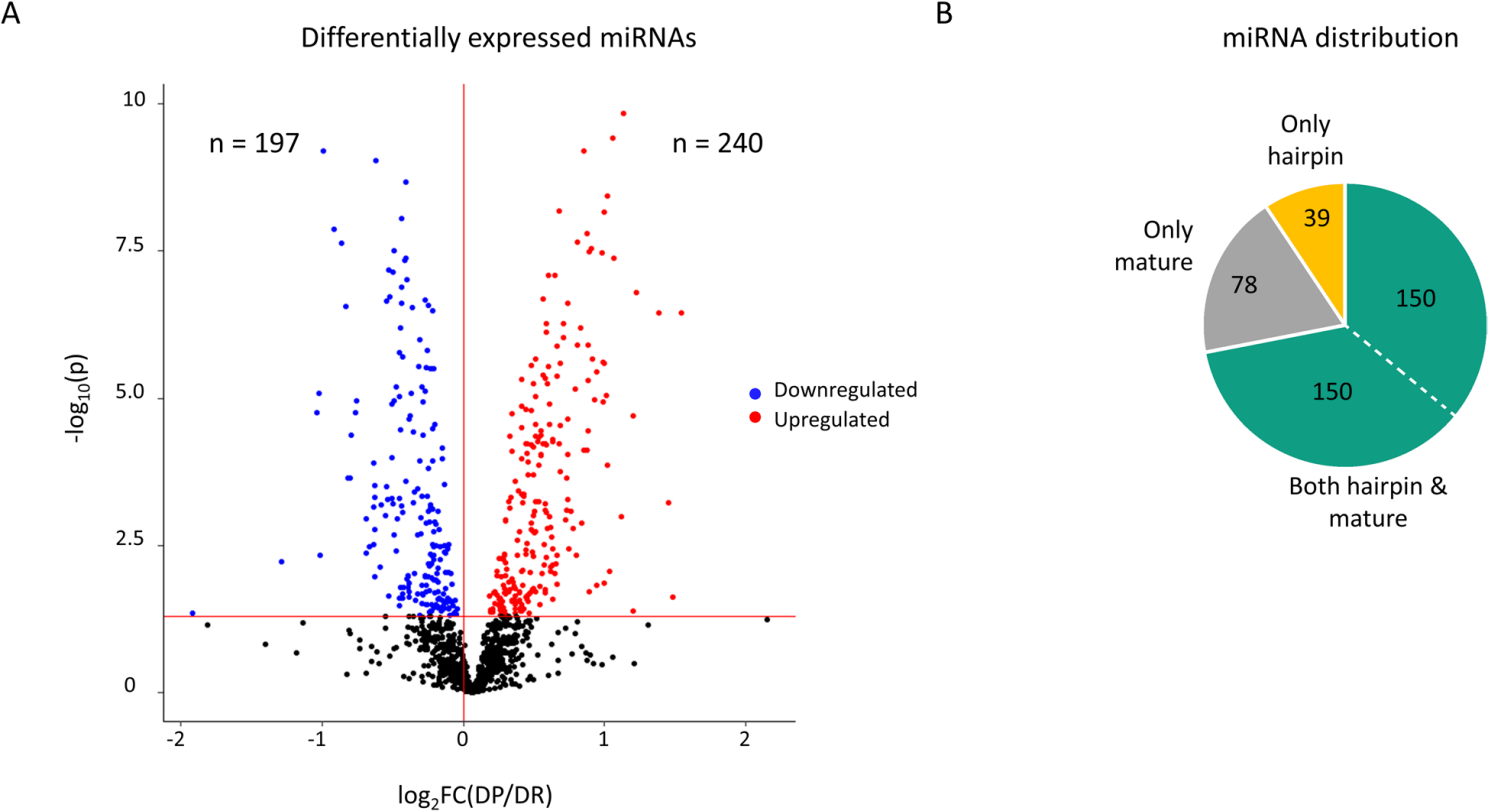
Adipose tissue miRNA profile predictive of T2D. **A** Volcano plot showing the fold change (*x*-axis) and *p*-value (*y*-axis) for all miRNAs. Blue = downregulated in DP; red = upregulated in DP; *n* = number of miRNAs; *p* < 0.05. **B** Pie chart with miRNAs description specifically showing gWAT-derived miRNAs with overlapping hairpin and mature miRNA profiles

Using the small RNA sequencing, a total of 437 miRNAs were found to be differentially expressed (*p* < 0.05) in the gWAT of DP mice compared to DR (Fig. 3A). Among these, 197 miRNAs were downregulated and 240 were upregulated in DP. Of note, these numbers include both mature and hairpin miRNAs. As miRNAs can be released from the expressing cells and taken up by different cell types [15], miRNA levels measured in the gWAT can be a mixture of both. Specifically, 150 miRNAs were found to be differentially expressed in DP mice (*p* < 0.05) and also have the corresponding hairpin miRNA following a similar pattern (Fig. 3B; Additional file 2: Table S1). Of note, 110 miRNAs among the 150 were also conserved in humans with at least 7 consecutive nucleotide position matches (Additional file 2: Table S2). The miRNA expression changes of four miRNAs identified by small RNA sequencing were also validated with RT-qPCR (Additional file 1: Fig. S2). Altogether, this data hints at an important role of miRNAs in defining the future risk of T2D.

### Dysregulated imprinted miRNAs in adipose tissue of diabetes-prone mice

**Fig. 4:**
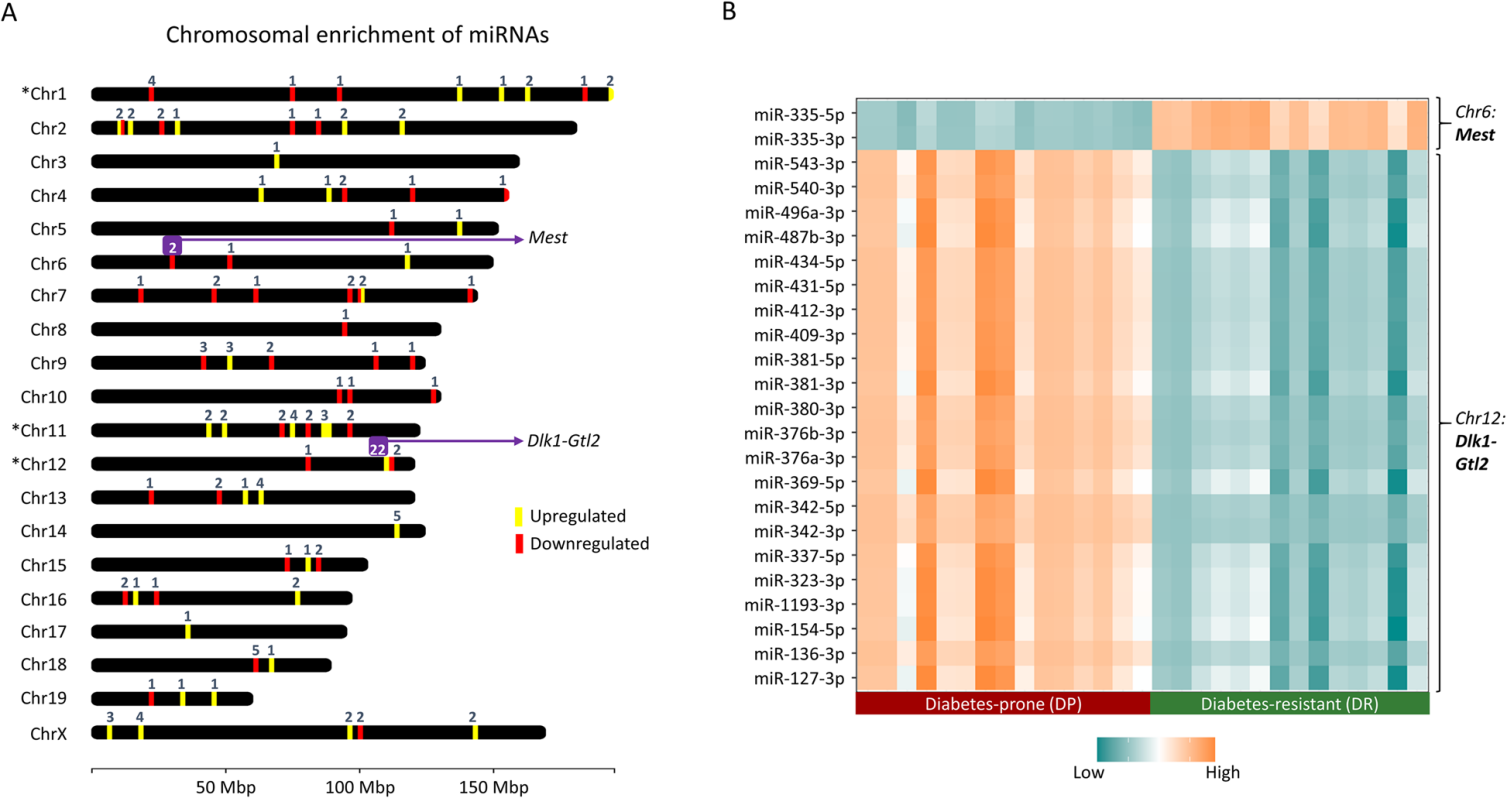
Chromosomal localization of differentially expressed miRNAs highlights imprinted miRNAs linked with T2D. **A** ChromoMap shows the differentially expressed miRNAs in the present study. Yellow tags correspond to upregulated miRNAs and red corresponds to downregulated ones in DP compared to DR; *p < 0.05 based on Fischer’s exact test. **B** The heatmap shows the mean expression of the imprinted miRNAs in DP and DR mice. The data are scaled and the colour bar corresponds to the higher or lower expression of a particular miRNA for the DP and DR group

The genomic distribution of the 150 mature differentially expressed miRNAs highlighted several miRNA clusters based on the chromosomal location. As shown in Fig. 4A, the differentially expressed miRNAs were distributed among all chromosomes; however, a significant enrichment (more than expected) of miRNAs was found for chromosomes 1, 11, and 12 (Fig. 4A and Additional file 1: Results). Interestingly, 22 miRNAs that cluster on chromosome 12 mapped to the imprinted genome region *Dlk1-Gtl2*, which is conserved across mice and humans and codes for more than 40 miRNAs within a 10 kb distance. All the miRNAs transcribed from this locus had an elevated expression in DP mice compared to DR, highlighting the potential epigenetic regulation (Fig. 4B). Not only this locus, but also another imprinted miRNA, miR-335 from the *Mest* locus on chromosome 6, was significantly downregulated in DP mice (Fig. 4B).

Apart from the imprinted miRNAs, a cluster of 5 miRNAs (miR-145a-5p/145a-3p/378a-5p/378a-3p/143-3p) was located on chromosome 18 and all were found to be downregulated, while another cluster of 5 miRNAs (miR-17-5p/17-3p/18a-5p/18-3p/19b-3p) located on chromosome 14 was found to be upregulated in the adipose tissue of DP mice. Next, we screened for transcription factor binding sites (TFBS) to check if any of these miRNA clusters also have a common regulatory transcription factor. For the imprinted miR-335, the miRNA transcription factor E2F3 was found to be significantly enriched; for the other loci, several miRNA-TF pairs were identified that could potentially regulate miRNA transcription (Table 1). E2F3 is the only E2F member that is linked to cancer [32] and is a potent and direct regulator of *Igf2*, with its role in adipose tissue still not clear. Of note, *E2f3* was found to be differentially expressed in the gWAT of DP mice (FC_DP/DR_ = 1.1; adjP = 0.034). For the large cluster on chromosome 14 (miR-17/18a/19b), transcription factor TGIF1 (Transforming growth factor-induced factor homeobox 1), and for the other cluster on chromosome 18 (miR-145a/378a/143-3p), NR2F6 (Nuclear receptor subfamily 2 group F member 6) was found to be enriched. TFs that were also found to be differentially expressed (e.g. *E2f3*, *Npas4*, *Tgif1*) in the adipose tissue of DP mice are highlighted in Table 1.

**Table 1:** Putative transcription factors regulating the imprinted miRNAs cluster. Transcription factor binding motif enrichment analysis in the promoter (500 kb upstream of transcription start site) of imprinted/clustered miRNAs

miRNA gene	Chr	Transcription factor (enriched)
miR-335	6	**E2F3**
miR-543 miR-540 miR-487b miR-434 miR-409 miR-323 miR-1193 miR-136	12	CREM, **NPAS4**, ZBED1 ZNF140 TP53 TBX6 GLIS1 **NFIB**, **NFIC**::TLX1 HES7 ZNF354C, CTCF, ZNF416, **ELF4**, ZNF213, THAP11, **ZBTB11**, **ELF1**, NRF1, **PLAGL1**, **GABPA**, ZFP42, MYCN, NR1D2
miR-145a/378a/143	18	NR2F6
miR-17/18a/19b	14	**TGIF1**

Data is based on an enrichment test

Double colon indicates a co-factor

Bold are also differentially expressed TF in the adipose tissue of DP versus DR mice

### WMCNA analysis highlights several known and some novel miRNAs linked to T2D

**Fig. 5:**
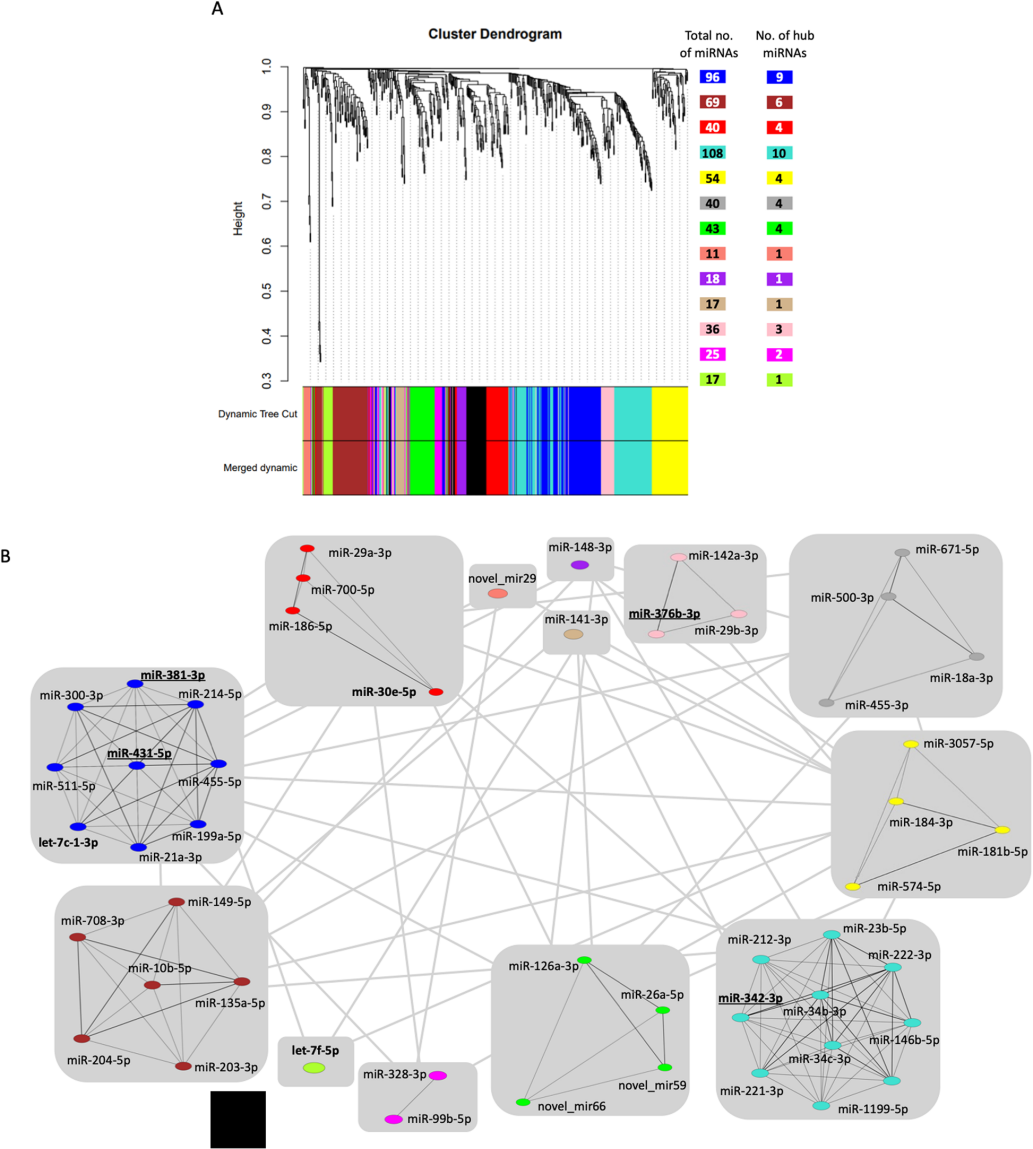
Weighted miRNA co-expression network analysis (WMCNA) with hub miRNAs. **A** Clustering dendrogram of miRNAs with module size. Each module is labelled with unique colour and the number besides corresponds to the number of miRNAs belonging to each module and number of hub miRNAs in each module. **B** Schematic representation of the module hub miRNAs and their inter- and intra-module interconnectedness shown as lines. The hub miRNAs either identified in previous studies to be linked with T2D or belonging to the imprinted loci (*Dlk1-Gtl2,* Mest) are in bold with underline

To enhance the understanding of miRNome regulation and to identify those miRNAs that may play a central role in T2D risk, weighted miRNA co-expression network analysis (WMCNA) was performed. The clustering identified 13 modules and no miRNA was left unassigned (Fig. 5A). The maximum number of miRNAs was assigned to the turquoise module (*n* = 108), followed by blue (*n* = 96) and brown (*n* = 69). For each module, the hub miRNAs were identified as shown in Fig. 5B. Interestingly, the miRNAs let-7c-1-3p, let-7f-5p, and miR-30c-5p, belonging to let-7 and miR-30 families, respectively, that have been previously identified to be associated with T2D in humans [20], were also among the hub miRNAs. Overall, among the 50 hub miRNAs identified by WMCNA (Fig. 5A and B), 35 were already linked to the pathophysiology of T2D (Additional file 1: Results). New candidates were miR-214-5p, miR-431-5p, miR-1199-5p, miR-300-3p, miR-511-5p, miR-181b-5p, miR-3057-5p, miR-700-5p, miR-186-5p, miR-500-3p, miR-671-5p, miR-328-3p, and three other novel miRNAs (novel_miR29, novel_miR59, and novel_miR66), which will be investigated in more detail in future studies. Of note, among these hub miRNAs were also four miRNAs (miR-381-3p, miR-431-5p, miR-376b-3p, and miR-342-3p) belonging to the imprinted cluster located on chromosome 12 (highlighted in Fig. 5B).

### Snapshot of miRNA-mRNA-protein map of adipose tissue from diabetes-prone mice

**Fig. 6:**
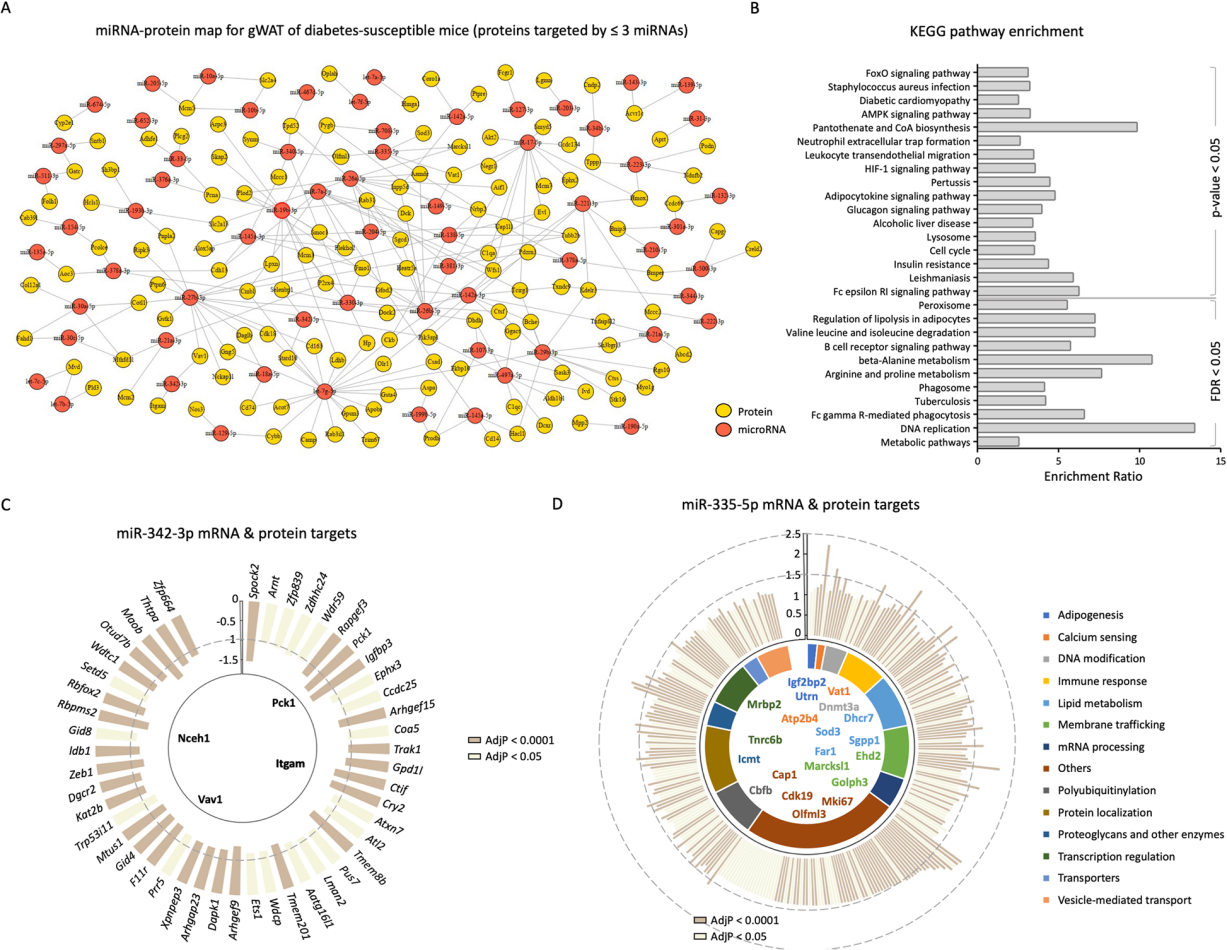
Snapshot of miRNA-mRNA-protein map of adipose tissue from diabetes-prone mice. **A** miRNA-protein network based on differentially expressed miRNAs and proteins that are also differentially expressed in gWAT of DP mice. Circles represent miRNA or protein and lines represent putative regulation for proteins putatively targeted by ≤ 3 miRNAs. **B** Bar plot with enrichment ratio (*x*-axis) for KEGG pathway enrichment analysis for 227 proteins. *Y*-axis maps to the pathways (*p* < 0.05 and FDR < 0.05). Integration of the miRNA-mRNA-protein targets for miR-342-3p **C** and miR-335-5p **D**. Circular bar plot shows the experimentally validated mRNA targets for each miRNA that are also differentially expressed in the gWAT of DP mice in the direction opposite to that of the miRNA. The innermost track shows the proteins that are potential targets for miRNAs and are also differentially expressed. The *y*-axis represents the fold change (DP/DR) and the colour of the bars is based on the *p* value (dark = AdjP < 0.0001; light = AdjP < 0.05)

Transcriptome and proteome profiles were integrated with the miRNA expression data to decipher the molecular mechanisms affected by the altered miRNAs in gWAT (Fig. 6A). The mRNA and miRNA expression were analysed in the same samples, thus allowing a robust analysis. Using the online databases, putative yet experimentally validated mRNA targets for all differentially expressed miRNAs were extracted (Additional file 2: Table S3). For the 150 differentially expressed miRNAs, a total of 9523 experimentally validated mRNA targets were identified. Among these, 5045 (upregulated 2591 and downregulated 2454) were differentially expressed in the gWAT of DP versus DR mice (AdjP < 0.05; Additional file 2: Table S3). Lastly, to evaluate if the gene expression changes also translate to the protein expression changes, differentially expressed proteins in gWAT of DP versus DR mice were integrated with the altered transcriptome. This analysis resulted in 227 genes that are affected on the mRNA level also to be potentially regulated at the protein level (Fig. 6A, Additional file 1: Fig. S3 and Additional file 2: Table S4) and were found to be enriched (*p* < 0.05) for pathways involved in the regulation of amino acid metabolism, inflammation, signalling, and insulin resistance (Fig. 6B).

Of note, these 227 proteins are putative yet experimentally validated targets of 84 miRNAs. As expected, one miRNA was able to target several mRNAs/proteins; for instance, miR-27b-3p may target 53 and miR-let-7 g-5p may target 49 proteins according to the current dataset. Among the 227 proteins, several are known to be involved in insulin sensitivity and lipid metabolism. For instance, insulin receptor substrate 1 (Irs1), solute carrier family 2 member 4 (Slc2a4, GLUT4), insulin-like growth factor 2 mRNA-binding protein 2 (Igf2bp2), and AKT serine/threonine kinase 2 (Akt2) are involved in the insulin cascade. Meanwhile, 7-dehydrocholesterol reductase (Dhcr7), apolipoprotein B receptor (Apobr), carnitine palmitoyltransferase 1a (Cpt1a), sterol O-acyltransferase 1 (Soat1), fatty acyl-CoA reductase 1 (Far1), oxidized low-density lipoprotein receptor 1 (Olr1), ATP-binding cassette, sub-family D member 2 (Abcd2), and patatin-like phospholipase domain containing 2 (Pnpla2) play an important role in lipid metabolic processes. Of note, protein levels of mesoderm-specific transcript protein (Mest), belonging to the AB hydrolase superfamily, were also different between the gWAT of DP and DR mice. This protein is targeted by six miRNAs: miR-let-7b-5p, miR-let-7c-5p, miR-143-3p, miR-27b-3p, miR-29b-3p, and miR-378a-3p. In addition, several proteins were targeted by more than 15 miRNAs, including utrophin (Utrn), associated with lipid expandability during adipogenesis, and proliferation marker protein Ki-67.

### Imprinted miRNAs target genes crucial for adipose function in diabetes-prone mice

Next, the mRNA targets of miRNAs belonging to *Dlk1-Gtl2* (of the 22 differentially expressed miRNAs, miR-342-3p was selected) and *Mest* (miR-335-5p) locus were screened for changes in gene expression profiles (Fig. 6C and D). For miR-342-3p, 161 putative mRNA targets were identified, and 47 of these were found to be downregulated in gWAT of DP mice (Fig. 6C). Of note, some important targets were *Cry2* (Cryptochrome circadian regulator 2), *Igfbp3* (insulin-like growth factor binding protein 3), and *Ets1* (ETS proto-oncogene 1, transcription factor). The overlap with the proteome, however, resulted in only four proteins with differential expression in the gWAT of DP mice: neutral cholesterol ester hydrolase 1 (Nceh1), phosphoenolpyruvate carboxykinase 1 (Pck1), Vav guanine nucleotide exchange factor 1 (Vav1), and Itgam (Integrin subunit alpha M).

A total of 1047 experimentally validated mRNA targets were retrieved from the in-house database for miR-335-5p. Among these, 291 mRNAs were found to be upregulated in the gWAT of DP mice compared to DR (Fig. 6D and Additional file 1: Fig. S5). The majority of these genes were found to be involved in membrane trafficking, vesicle-mediated transport, protein localization, immune response, lipid metabolism, and adipogenesis. Furthermore, the overlap with differentially expressed proteins resulted in the identification of 20 putative targets, including plasma membrane calcium-transporting ATPase 4 (Atp2b4) and synaptic vesicle membrane protein VAT-1 homolog (Vat1) involved in calcium sensing; Dhcr7, Far1, protein-S-isoprenylcysteine O-methyltransferase (Icmt), sphingosine-1-phosphate phosphatase 1 (Sgpp1), extracellular superoxide dismutase (Sod3) in lipid metabolism; Igf2bp2 and Utrn in adipogenesis.

### Almost half of the differentially expressed miRNAs in gWAT are under epigenetic control

As changes in miRNA expression are closely associated with altered DNA methylation [20, 33, 34], the DNA methylation profiles for the differentially expressed miRNA genes were investigated. Out of 150 miRNAs, 70 also had differences (*p* < 0.05) in at least one CpG site annotated to the miRNA gene when comparing DP versus DR mice (Additional file 2: Table S5). The most prominent changes were in the CpG site, cg29882819, annotated to the miR-21a gene (|%| change in DNA methylation = 11.3%; *p* = 2.8 × 10^−8^), and cg33631853 annotated to miRNA-let-7c-2/7b (|%| change in DNA methylation = 8.8%; *p* = 1.73 × 10^−6^). As the imprinted miRNAs are under monoallelic regulation and epigenetically controlled by the DNA methylation status of the imprinting control region (ICR), the DNA methylation changes in the ICR for these miRNAs were also investigated. For the miRNAs belonging to the *Dlk1-Gtl2* locus, out of eight experimentally validated CpGs based on the MM285 array, seven were found to be differentially methylated. However, for miR-335/*Mest*, two CpG sites were found to be differentially methylated (Table 2).

**Table 2:** DNA methylation of specific CpGs annotated for the imprinted miRNAs

Locus/ gene	CpG ID	Chr	Average DNA methylation ± SD (DP)	Average DNA methylation ± SD (DR)	|%| change	*p*	miRNAs
*Mest*	cg43264716_BC21	6	0.522 ± 0.02	0.494 ± 0.02	2.75	0.007828	**miR-335-5p**; miR-335-3p
*Mest*	cg43264721_BC21	6	0.393 ± 0.02	0.416 ± 0.02	2.33	0.040658	
*Dlk1-Gtl2*	cg31119458_TC21	12	0.699 ± 0.01	0.643 ± 0.01	5.64	3.7E-07	miR-543-3p; miR-540-3p; miR-496a-3p; miR-487b-3p; miR-434-5p; miR-431-5p; miR-412-3p; miR-409-3p; miR-381-5p; miR-381-3p; miR-380-3p; miR-376b-3p; miR-376a-3p; miR-369-5p; miR-342-5p; **miR-342-3p**; miR-337-5p; miR-323-3p; miR-1193-3p; miR-154-5p; miR-136-3p; miR-127-3p
*Dlk1-Gtl2*	cg31119465_BC21	12	0.505 ± 0.02	0.451 ± 0.02	5.36	0.000483	
*Dlk1-Gtl2*	cg31119460_TC21	12	0.558 ± 0.01	0.526 ± 0.01	3.20	0.000718	
*Dlk1-Gtl2*	cg31119462_TC21	12	0.520 ± 0.01	0.500 ± 0.01	2.07	0.01158	
*Dlk1-Gtl2*	cg31119459_TC21	12	0.798 ± 0.02	0.775 ± 0.02	2.29	0.021666	
*Dlk1-Gtl2*	cg31119463_TC21	12	0.457 ± 0.02	0.434 ± 0.02	2.30	0.025192	
*Dlk1-Gtl2*	cg31119469_BC21	12	0.271 ± 0.01	0.252 ± 0.02	1.92	0.033323	

Data is based on beta (β) values obtained from the MM285 Illumina mouse array

|%| change Absolute percentage change in DNA methylation of DP and DR

*DP *Diabetes-prone, *DR *Diabetes-resistant

p- *p* value

### Ortholog miRNAs were similarly altered in monozygotic twin pairs discordant for T2D

In the attempt to translate our results to humans, mouse miRNome data were compared to miRNA profiles from the adipose tissue of monozygotic twin pairs discordant for T2D [20, 29]. As mentioned earlier, the currently used isogenic mouse model is ideal for capturing the underlying epigenetic mechanisms driving T2D. Thus, adipose tissue from our twin study is appropriate for translating these findings [29]. We previously analysed the expression of 408 miRNAs known to be expressed in adipose tissue and described 30 miRNAs to be different between monozygotic twin pairs discordant for T2D [20]. In the present study, among the 150 differentially expressed miRNAs detected in mice, 110 were conserved in humans, and those were explored in the monozygotic twin pairs discordant for T2D.

Of these, 100 miRNAs were detected by the array used for the original study [20], and 12 of these miRNAs were differentially expressed in the discordant twin pairs (*p* < 0.05; Additional file 2: Table S6). One newly identified candidate was miR-378g (corresponding to miR-378a-3p in mice), which was found to be down-regulated in both T2D monozygotic twins and DP mice (fold reduction = 1.3). The miRNAs were also correlated with the fasting plasma glucose levels and HbA1c levels (Additional file 2: Table S7). Expression levels of 378a-3p correlated negatively with fasting plasma glucose levels (*ρ* = − 0.451; *p* = 0.027) in the monozygotic twin pairs discordant for T2D. Another miRNA, miR-146b-5p, which was identified as one of the hub miRNAs in the murine model, correlated positively with HbA1c (ρ = 0.427; *p* = 0.038).

**Fig. 7:**
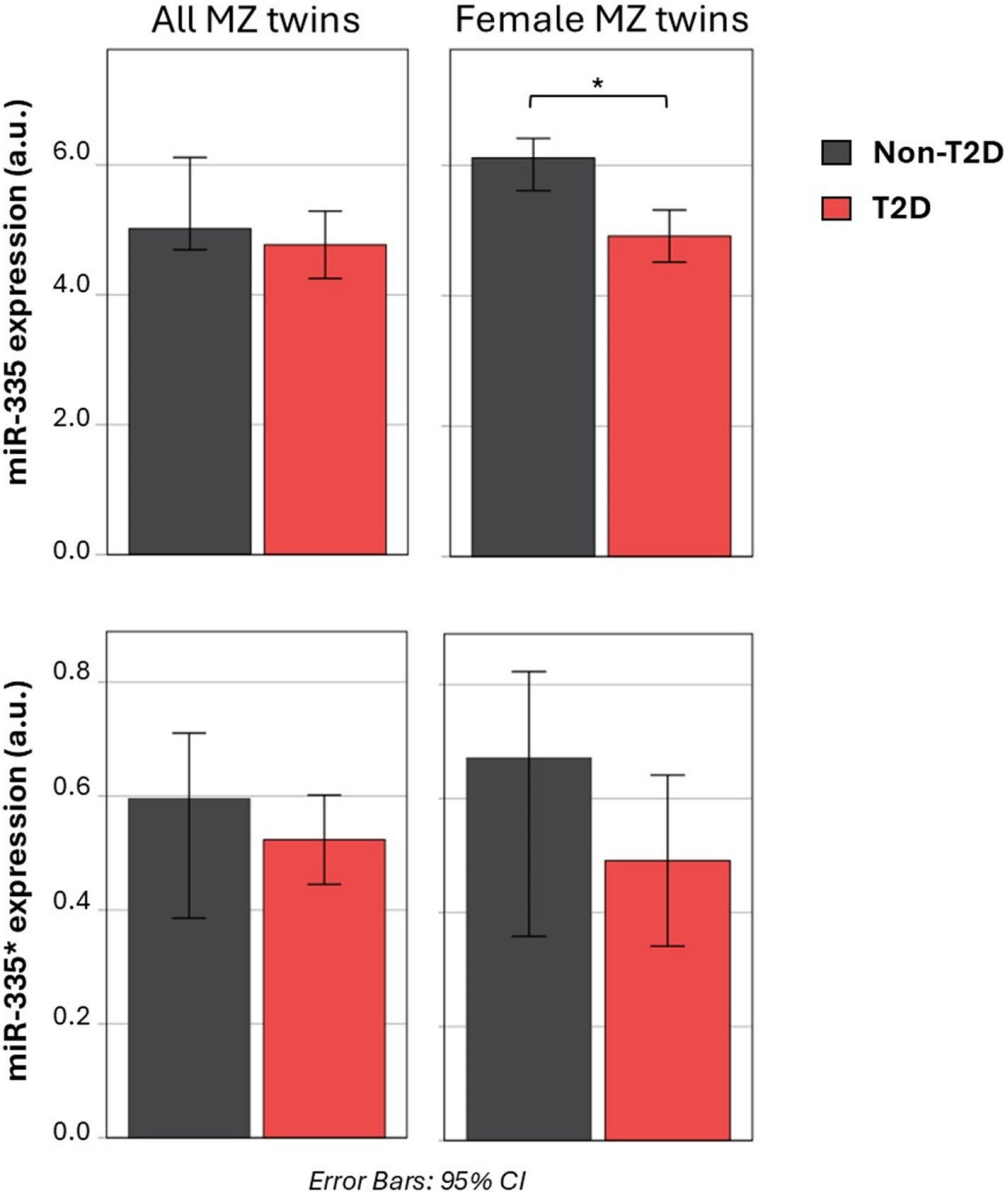
Difference in miR-335 expression in adipose tissue of female monozygotic twin pairs discordant for T2D. Bar plots of the medians of miR-335 and miR-335* (* miR-335-3p according to miRbase v17) expression values in adipose tissue according to the different groups (i.e. non-T2D and T2D) of the monozygotic twin pairs discordant for T2D (N pairs = 12) and the subgroup of female twin pairs (N pairs = 5). Expression of miRNA-335 was found to be decreased in adipose tissue from T2D female twins compared with co-twins without T2D (N pairs = 5). Error bars with 95% CI are shown. *p < 0.05 at the paired two-tailed Wilcoxon test. a.u., arbitrary units; MZ, monozygotic; CI, confidence interval

**Fig. 8:**
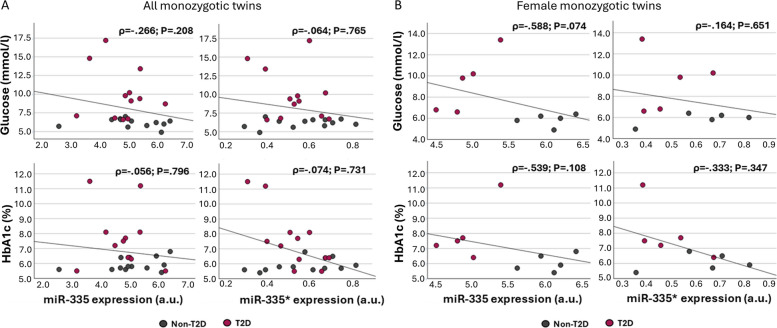
Adipose miR-335 expression shows negative correlation trend with metabolic readouts only in female twin pairs. **A** Scatter plots showing the correlation between miR-335 and miR-335* expression values in adipose tissue and fasting plasma glucose (mmol/l) and HbA1c (%) from the monozygotic twin pairs discordant for T2D (N pairs = 12) and **B** the subgroup of female twin pairs (N pairs = 5). Spearman’s rank correlation coefficient (*ρ*) and *p* value of the Spearman statistics are displayed for each test

Interestingly, apart from the changes in the let-7 family (let-7f/7g/7a/7b) and miR-30 family (miR-30a/30c/30d) [20], imprinted miRNAs identified in the murine model were also found to be altered similarly in humans. The miR-342-3p was up-regulated in T2D, which was already identified in our previous twin study [20]. In the present study, we found that miR-342-3p correlated positively with HbA1c (*ρ* = 0.496; *p* = 0.014). For miR-335, we did not observe a different abundance when male and female human data were combined. However, there was a trend of reduced expression in T2D (Fig. 7). As the mouse screening was performed in female mice, we also considered the subgroup of 5 pairs of female monozygotic twins and identified a significant down-regulation of miR-335 in T2D (*p* = 0.043; Fig. 7). Accordingly, a negative correlation trend was observed between miR-335 and fasting plasma glucose levels in female twins (*ρ* = − 0.588; *p* = 0.074) (Fig. 8). These results indicate that miR-335 is a female-specific marker for T2D. We also checked for intra–twin pair correlations between within–twin pair differences of the miRNAs above, and they were not significant (data not shown).

As we have observed that more than 50% of the differentially expressed miRNAs in mice also exhibit differences in DNA methylation, we compared the 70 differentially methylated CpGs identified in mice with the corresponding human sites (Additional file 2: Table S8). Among these, only cg08761490 (MIR335; MEST) and cg19019198 (MIRLET7G; WDR82) were differentially methylated in the discordant twins based on *p* < 0.05 (Additional file 2: Table S8), as previously reported [20, 29]. Correlation analysis revealed 12 CpG sites to be correlated with HbA1c levels based on *p* < 0.05 (Additional file 2: Table S9). Among these, four CpGs were annotated for the miRNAs belonging to the *DLK1-DIO3* (*Dlk1-Gtl2* in mouse) locus: cg14007694 (miR-369), cg02044725 (miR-487b), cg21876806 (miR-411), and cg13995230 (miR-376b). The cg08761490 annotated to the imprinted miRNA-335 also correlated with HbA1c (*ρ* = 0.475; *p* = 0.011) (Additional file 2: Table S9). Of note, three hub miRNAs identified in the mouse model using WMCNA, miR-26a (cg04787317), miR-376b (cg13995230), and miR-199a (cg24002149 and cg17178220) were also among these. Considering intra–twin pair correlations between within–twin pair differences of these CpG sites above, only cg13995230 and cg21876806 were respectively correlated with glucose and both glucose and HbA1c (*p* < 0.05) (data not shown).

#### miR-335-5p negatively correlates with fasting blood glucose levels in TÜF study

**Fig. 9:**
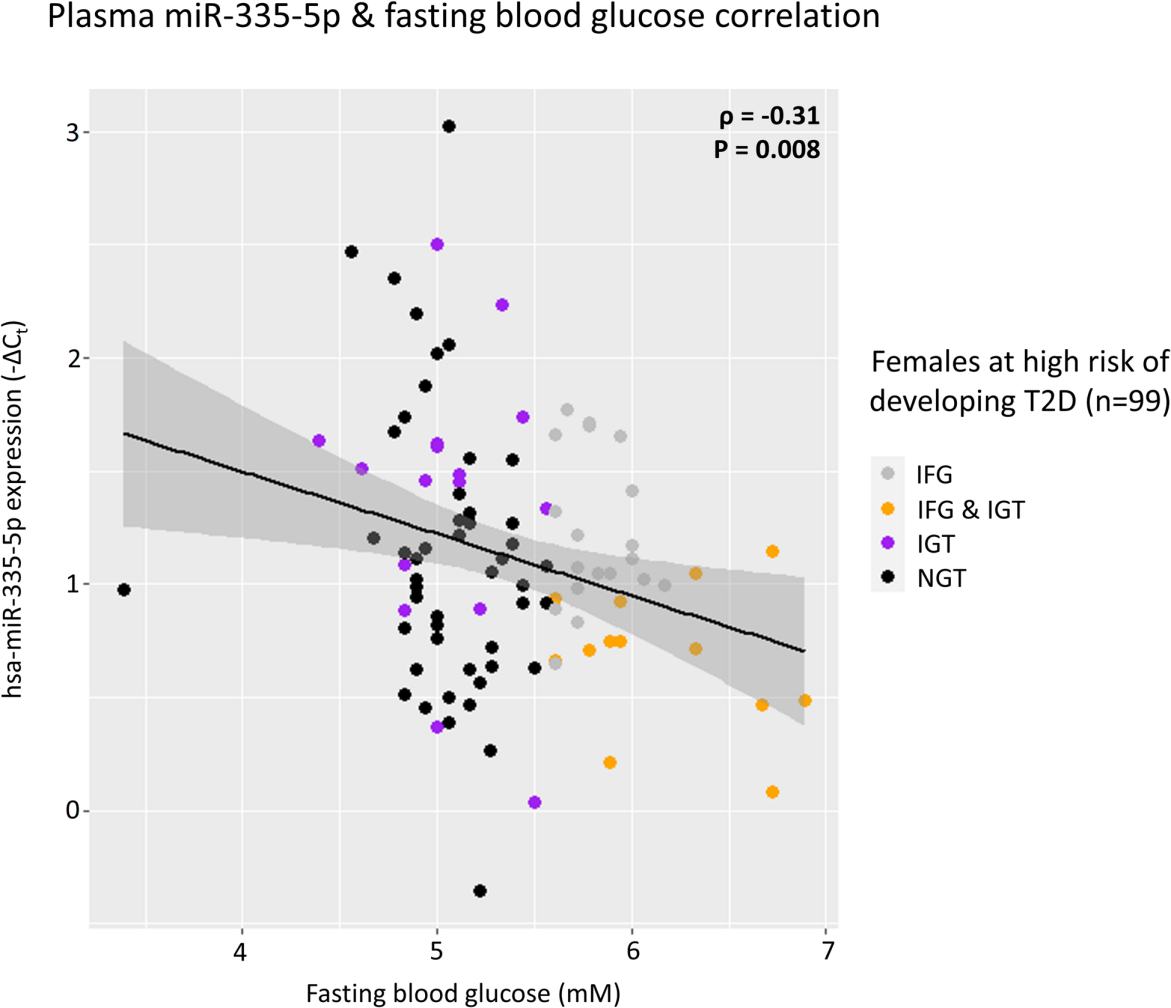
Plasma miR-335-5p negatively correlates with blood glucose in females at high risk of developing T2D. The plot shows the correlation between hsa-miR-335-5p plasma expression levels (*y*-axis) and fasting blood glucose levels (*x*-axis). Spearman’s rank correlation coefficient (*ρ*) and *p*-value of the Spearman statistics are displayed for each test. NGT, normal glucose tolerance; IGT, impaired glucose tolerance; IFG, impaired fasting glucose

Lastly, the expression level of miR-335-5p, which is conserved in humans and known to be secreted from the adipose tissue via extracellular vesicles [15], was investigated in 99 females at high risk of developing T2D based on elevated fasting glucose concentrations, a sub-cohort of the TÜF study [30]. The miRNA expression levels were measured in plasma along with fasting blood glucose levels and other anthropometric measurements. The miR-335-5p expression levels were found to be negatively correlated with the fasting blood glucose levels (*p* < 0.01), corroborating the findings in female diabetes-prone NZO mice and female pairs of monozygotic twins with T2D (Fig. 9). The same applies if only females with a body mass index > 26 were included in the analysis (*ρ* = − 0.30; *p* = 0.01). In conclusion, using a translational integrative approach, miR-335-5p was identified as a relevant miRNA linked to a potential biomarker for elevated risk of developing T2D, specifically in females.

#### Functional validation for miR-335-5p using 3T3-L1 cells

**Fig. 10:**
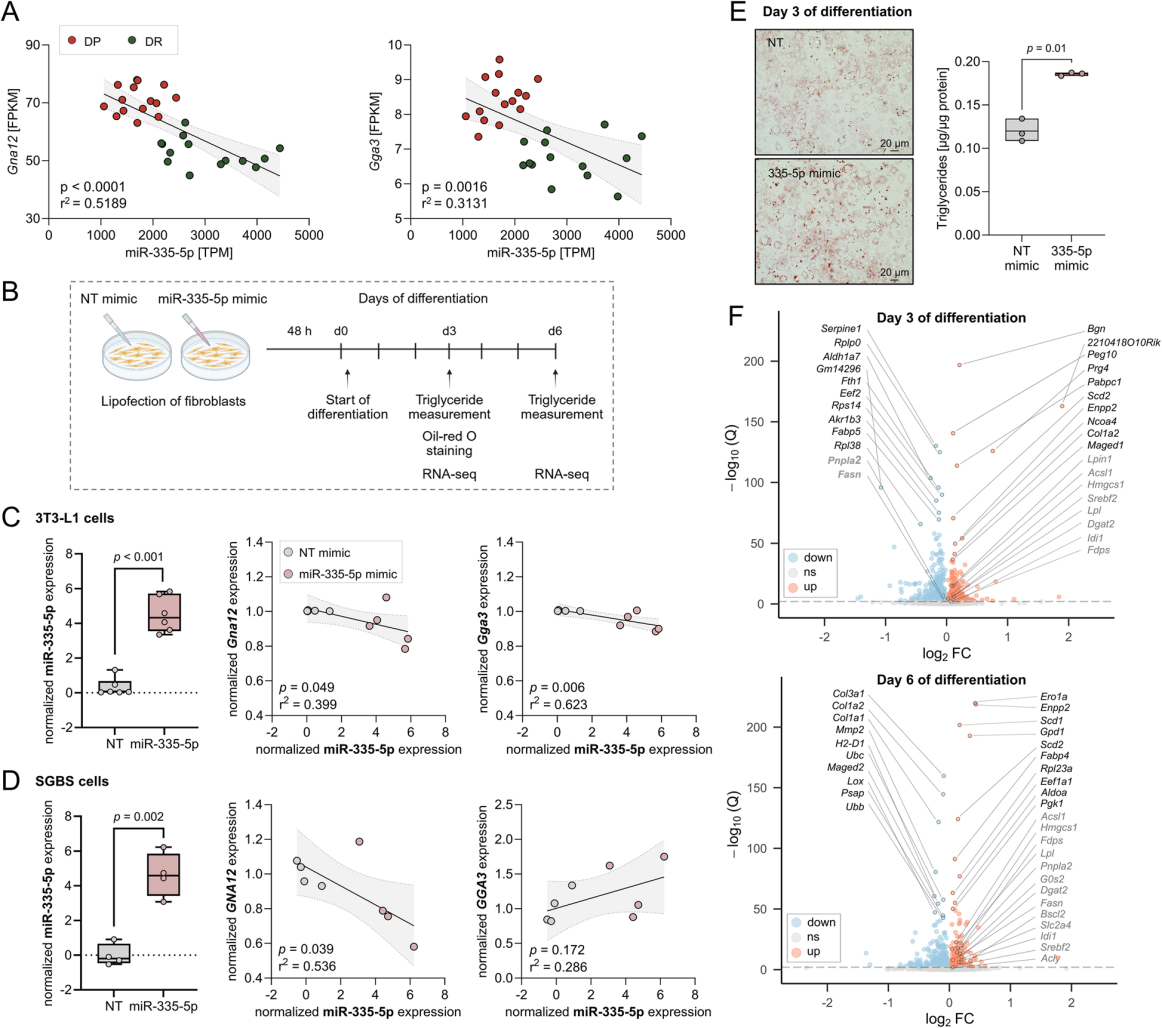
Molecular impact of miR-335-5p during 3T3-L1 differentiation and link between miR-335 and *GNA12* and *GGA3* in SGBS cells. **A** Correlation plots between the expression of putative genes of interest and miR-335-5p based on RNA and small-RNA sequencing, respectively. **B** Schematic overview of the experimental workflow. **C** Effect of miR-335-5p mimic transfection in 3T3-L1 fibroblasts and its correlation with *Gna12* and *Gga3* expression. **D** Effect of miR-335-5p mimic transfection in SGBS fibroblasts and its correlation with *GNA12* and *GGA3* expression. **E** Oil Red O staining of 3T3-L1 cells at day 3 of differentiation following transfection with NT mimic (upper panel) or miR-335-5p mimic (lower panel), along with quantification of intracellular triglyceride concentration. **F** Volcano plots of RNA sequencing data comparing NT mimic- and miR-335-5p mimic-transfected 3T3-L1 cells at day 3 (upper panel) and day 6 (lower panel) of differentiation. Top 10 up-/downregulated genes are highlighted in black; *q* < 0.01. Pearson correlation (*r*^2^) and corresponding *p*-values are displayed (**A**, **C** and **D**); Data represent the mean of three to six independent experiments (**C**, **D**, and **E**); unpaired *t*-test with Welch correction (**C**, **D**, and **E**). FPKM, fragments per kilobase of transcript per million mapped reads; TPM, transcripts per million

Based on the results from the mice and human data, we can conclude that reduced expression of miR-335 in the adipose tissue is associated with increased T2D risk, and this effect could be mediated by targeting specific genes. The genes to be tested in vitro in 3T3-L1 cells were selected based on the correlation between the miRNA and putative mRNA expression as detected by small-RNA and RNA sequencing, respectively (Fig. 10A).

Among these, *Gna12* (G protein subunit alpha 12) and *Gga3* (Golgi-associated, gamma-adaptin ear-containing, ARF-binding protein 3), both involved in membrane trafficking, were prioritized for validation due to their strong negative correlation with miR-335-5p expression. Transfection of 3T3-L1 fibroblasts with a miR-335-5p mimic (Fig. 10B) reduced the expression of *Gna12* and *Gga3*, showing a strong inverse correlation between miR-335-5p levels and target gene expression (*Gna12*: *p* = 0.049, *r*^2^ = 0.399; *Gga3*: *p* = 0.006, *r*^2^ = 0.623) (Fig. 10C). Consistent with these findings, transfection of human SGBS cells with the miR-335-5p mimic also led to reduced *GNA12* expression and a significant correlation (*p* = 0.039, *r*^2^ = 0.536), whereas *GGA3* showed no significant effect (*p* = 0.172, *r*^2^ = 0.286) (Fig. 10D).

To confirm the interaction between miR-355 and *Gna12* as well as *Gga3,* luciferase reporter assays were conducted in HeLa cells. As shown in Additional file 1: Fig. S4, co-transfection experiments revealed that miR-335 mimic significantly decreased luciferase activity levels through the binding sites of *Gga3* (20%, *p*-value = 1.2 × 10^−3^) when compared to the non-targeting mimic. However, the luciferase activity remained unchanged in cells transfected with *Gna12* and miR-335 mimic (Additional file 1: Fig. S4).

During 3T3-L1 differentiation, significant correlations for both target genes were observed on day 1 and day 5 upon overexpression of miR-335 (Additional file 1: Fig. S5A–B). Furthermore, miR-335-5p mimic treatment elevated triglyceride accumulation on day 3 of adipogenesis (Fig. 10E), whereas no effect was observed in mature adipocytes at day 6 (Additional file 1: Fig. S5C). Finally, insulin sensitivity as detected by pAkt in mature adipocytes revealed a tendency towards increased insulin sensitivity upon miR-335-5p overexpression (Additional file 1: Fig. S5D). To elucidate the molecular mechanisms underlying the increased triglyceride accumulation observed on day 3 of differentiation in 3T3-L1 cells treated with the miR-335-5p mimic, RNA sequencing was performed at days 3 and 6 of differentiation (Fig. 10F). On day 3, the top 10 up- and downregulated genes suggested activation of a transcriptional programme supporting early triglyceride accumulation. Among the downregulated genes were *Fabp5*, *Pnpla2*, and *Fasn*, which are involved in lipid metabolism. Specifically, *Scd2* and *Enpp2*, playing a role in fatty acid synthesis, were upregulated, presumably mediated in an indirect manner by miR-335. In addition, a marked reduction in ribosomal gene expression and *Fth1* was observed, along with induction of extracellular matrix (ECM)-related genes, including *Col1a2*, *Bgn*, and *Prg4*. By day 6, the expression profile of miR-335 overexpressing cells shifted towards an increased adipocyte maturation, characterized by increased expression of *Scd1*, *Scd2*, *Gpd1*, and *Fabp4*, with upregulation of glycolytic enzymes (*Aldoa* and *Pgk1*). In contrast, genes associated with ECM remodelling, including *Col1a1*, *Col1a2*, *Col3a1*, *Lox*, and *Mmp2*, were downregulated. Notably, expression of *Slc2a4*, although not among the top 20 differentially expressed genes, was also increased and confirmed at the protein level (Additional file 1: Fig. S5E). Taken together, these findings indicate that miR-335 acts as a potent molecular regulator, fine-tuning the expression of genes implicated in adipocyte triglyceride accumulation and an enhanced adipocyte maturation.

## Discussion

Using the NZO female mouse model, which allows to distinguish between diabetes-prone and -resistant mice weeks before the onset of T2D, early changes in the adipose tissue miRNA expression profile linked to ~ 5000 differentially expressed mRNAs and to 227 differentially abundant proteins were identified. Among the altered miRNAs were imprinted miRNAs belonging to *Dlk1-Gtl2* (22 miRNAs including miR-342-3p) and *Mest* (miR-335-5p) loci, which were upregulated and downregulated in diabetes-prone mice, respectively. WMCNA analysis revealed several known and novel hub miRNAs with an integrative miRNA-mRNA-protein snapshot highlighting molecular targets involved in pathways important for amino acid metabolism, insulin resistance, lipid metabolism, inflammation, and signalling. Specifically, the imprinted miRNA candidate, miR-335-5p target genes identified in the present study are related to processes such as adipogenesis, vesicle-mediated transport, and membrane trafficking. Moreover, the expression of miR-342-3p was altered in the adipose tissue of human twin pairs discordant for T2D in a similar manner, with miR-335 identified only in female twin pairs. The fact that circulating miR-335-5p abundance also negatively correlated with fasting blood glucose levels in female individuals at high risk of developing T2D indicates that this miRNA is a valuable marker for identifying prediabetic women who could benefit from preventive measures such as lifestyle changes.

Adipocytes are responsive to insulin and regulate whole-body nutrient homeostasis in a highly tuned manner, and adipose tissue insulin resistance is an important cue in deciphering the metabolic milieu underlying T2D pathogenesis [35]. Adipose tissue of diabetes-prone mice was insulin resistant based on the adipo-IR index and downregulation of key genes involved in insulin signalling. It is important to note that these changes are independent of changes in adipose tissue weight. A similar deterioration of adipose tissue function was also described previously in humans, where adipocyte insulin sensitivity was reduced in individuals with metabolic syndrome, with prominent alterations in the lipolysis cascade along with marked downregulation of the *IRS2* gene expression [36]. Also, the expression of *Slc2a4* (GLUT4), which not only transports glucose but also glucosamine and dehydroascorbic acid into the adipocytes, is a major readout for adipose tissue insulin sensitivity [37]. The expression of these two genes was reduced in the diabetes-prone mice, confirming an impaired adipose tissue insulin sensitivity already at week 10, way before the onset of T2D.

MiRNAs are key post-transcriptional regulators that modulate cellular functions by binding to complementary sequences in target mRNAs, primarily within the 3' UTR, leading to translational repression/mRNA degradation [38]. The miRNAs can also interact with the 5' UTR, coding regions, or promoter sites, influencing gene expression while their expression is tightly controlled by epigenetic mechanisms. In addition, miRNAs can regulate epigenetic enzymes, forming feedback loops that affect chromatin dynamics [39]. MiRNAs can be secreted and transferred between cells via extracellular vesicles, allowing them to function as intercellular messengers [40]. These multi-layered regulatory mechanisms enable miRNAs to play a central role in maintaining cellular homeostasis and responding to environmental and physiological changes [41]. Epigenetic alterations such as DNA methylation changes or miRNA expression are also linked with adipose tissue dysfunction in mice and humans. These can occur in response to adverse environmental factors including undernutrition during early development, thereby predisposing certain individuals to the risk of developing cardiometabolic diseases including adiposity and/or T2D. Our work focused on the aspect of predisposition, and we asked whether early alterations in the adipose tissue miRNome could indeed be the molecular component predisposing individuals to T2D risk. The fact that members of the let-7 and miR-30 families, known to play an important role in human T2D [20], were identified in our study clearly shows that the NZO model is appropriate to discover novel miRNAs contributing to the pathophysiology of T2D. Although several studies investigated the role of miRNAs in obese and diabetic mice [42, 43], this is the first study providing an adipose tissue-specific snapshot of molecular alterations before the onset of T2D.

Our combinative approach in the present study suggests a link between miRNA and DNA methylation, specifically unfolding the molecular importance of imprinted miRNAs belonging to *Dlk1-Gtl2* (22 miRNAs including miR-342) and *Mest* (miR-335) loci in modulating the response to caloric stress. In obesity, imprinted miRNAs can influence the development and function of adipose tissue, which is essential for storing fat, regulating energy balance, glucose metabolism, and insulin signalling pathways [20]. Expression of all miRNAs belonging to the *Dlk1-Gtl2* locus was upregulated in diabetes-prone mice, while one of the most highly expressed miRNAs, miR-342-3p (a hub miRNA), was also increased in monozygotic twins with T2D [20]. On the other hand, miR-335-5p expression was reduced in female mice prone to develop T2D and in female twins with T2D. Epigenome editing of the *Dlk1-Gtl2* imprinted control locus has confirmed DNA methylation as a crucial component in the transcription of this locus [44].

In the present study, the DNA methylation of both of these imprinting control loci was altered in diabetes-prone mice. Genomic disruption of the imprinted locus on chr12 in mice is known to result in severely dysfunctional energy homeostasis with marked hypoglycemia [45]. MiR-342-3p expression correlated positively with the HbA1c levels in monozygotic twin pairs discordant for T2D, in line with a previous study where miR-342 was also found to be upregulated in visceral adipose tissue of individuals grouped as prediabetic or diabetic compared to normoglycemic [46]. Importantly, recently in two independent INNODIA cohorts, the expression of three miRNAs (miR-409-3p, miR-127-3p, and miR-382-5p) belonging to the same locus was identified to be sufficient in stratifying two sub-clusters of individuals with recent-onset type 1 diabetes [24]. Altogether, these findings propose novel mechanisms related to metabolic imprinting in the context of T2D.

DNA methylation of specific CpG sites annotated to miR-335 correlated negatively with the HbA1c levels in the present study. In addition, a novel miRNA, miR-378g, was identified to be linked with T2D in both murine model and twin pairs. It is important to note that Can et al. have previously profiled the circulating miRNA levels in 45 obese children and adolescents and 41 age-matched lean controls, identifying a striking reduction in miR-335 and an elevation of miR-378 expression. Moreover, miR-335 levels were positively correlated with adiponectin and high-density lipoprotein cholesterol levels and negatively with leptin, triglycerides, body mass index, and HOMA-IR [47]. Specifically, miR-335-5p is a multifunctional miRNA involved in the regulation of diverse biological processes, including metabolism, cell proliferation, inflammation, and neuronal function in several cell types [48–50]. In the context of metabolic regulation, miR-335-5p has been shown to influence key pathways related to glucose homeostasis and insulin sensitivity, including the modulation of genes such as *SIRT1*, *PPARG*, and components of the PI3K/Akt pathway [49, 51, 52]. Its expression is responsive to metabolic stress and may contribute to adipocyte differentiation and lipid accumulation [53]. Notably, in models of T2D, miR-335-5p has been implicated in pancreatic β-cell dysfunction and impaired insulin secretion through its regulation of pro-apoptotic targets [54]. Additionally, miR-335-5p modulates inflammatory signalling pathways, including the suppression of cytokine regulators, linking it to immune regulation and oxidative stress responses [52, 55]. Our functional in vitro characterization of miR-335-5p using mimics suggests a shift in the programme of adipogenesis. The gene expression changes observed on day 3 indicate the activation of a transcriptional programme that promotes early triglyceride accumulation in an immature adipogenic context. Upregulation of *Scd2* and *Enpp2* likely enhances monounsaturated fatty acid (MUFA) synthesis and lysophosphatidic acid signalling, while downregulation of *Aldh1a7* (a member of the Aldh1 family) and *Fabp5* suggests a release of retinoic acid-mediated suppression and reduced fatty acid shuttling into oxidative pathways. Concurrent decreases in ribosomal genes and *Fth1* point to a stress-associated transitional state, whereas induction of extracellular matrix (ECM)-related genes (*Col1a2*, *Bgn*, *Prg4*) indicates active tissue remodelling. By day 6, the transcriptional profile shifts towards a classical adipocyte maturation programme, supporting balanced lipid homeostasis and improved metabolic flexibility. Increased expression of glycolytic enzymes (*Aldoa*, *Pgk1*) points towards enhanced glycolytic flux, providing both ATP for insulin signalling cascades and intermediates for de novo lipid synthesis. In parallel, decreased expression of ECM-related genes and upregulation of *Slc2a4*/GLUT4 likely underlie the modest improvement in insulin sensitivity, pointing to a mechanism that promotes enhanced glucose uptake and utilization.

Circulating miRNAs provide a unique opportunity to use them as potential biomarkers. For instance, three circulating miRNAs, miR-181b-5p, miR-1306-3p, and miR-3138, were highly accurate in discriminating individuals with elevated visceral adipose tissue inflammation [56]. Several other miRNAs have been identified to be derived from adipose tissue, packed into extracellular vesicles, and potentially regulating target genes in distant tissues, such as miR-222 linked with changes in IRS1 expression in liver and skeletal muscle [57]. Reduced plasma miR-223-3p was linked with adipose tissue insulin sensitivity index in another large cohort [42]. Our study design, using an isogenic mouse model and monozygotic twin pairs, offers a unique opportunity to control for potential confounders such as differences in genetic background, early-life environmental exposure, and age. However, the circulating miRNAs were investigated in female individuals at high risk of developing T2D, leading to the identification of miR-335-5p to play a role in early prediabetes specifically in females. However, today it is too early to establish miR-335-5p as a biomarker for clinical application without further studies in other human cohorts. While this study is largely based on mouse models, the conserved expression patterns observed in our small human cohort suggest possible relevance across species. In a few large cohort studies like the Framingham Heart Study, miR-335-5p levels were associated with cardiometabolic traits, including BMI and fasting insulin [58]. In patients with fatty liver, miR-335-5p was found to be dysregulated and linked to hepatic lipid accumulation and steatosis severity, and elevated levels have also been reported in obese children with insulin resistance [59, 60]. However, future work will explore how miR-335-5p expression evolves during the transition from normoglycemia to insulin resistance and T2D, ideally in longitudinal human cohorts. In addition, future mechanistic studies to clarify the functional importance, diagnostic, and therapeutic potential are required.

## Conclusions

In summary, an integrative translational approach including NZO female mice and twin pairs discordant for T2D led to the identification of early changes in adipose tissue miRNome linked to T2D susceptibility. Mapping the miRNA-mRNA-protein networks resulted in the identification of several novel molecular targets involved in adipose tissue function. The alteration of miRNAs from imprinted loci, along with changes in the DNA methylome, suggests a major contribution of epigenetic mechanisms in the development of T2D, proposing miR-335-5p as a potential biomarker which in the future may be useful for early detection and prevention of T2D in women.

## Supplementary Information

Additional File 1: Methods. Expression proteomics analysis. Western blot analysis in 3T3-L1 cells. miRCURY assay-based validation for selected miRNAs & gene expression. Validation of GNA12 and GGA3 in SGBS cells. Demographic information of the human cohorts. Results. Chromosomal enrichment analysis for 150 differentially expressed miRNAs. Weighted miRNA co-expression network analysis- hub miRNAs & T2D. Figures S1-S5. Fig S1- Plasma insulin and free fatty acid levels. Fig S2 – miRCURY assay-based qPCR validation of selected miRNAs. Fig S3 – Snapshot of miRNA-mRNA-protein map of adipose tissue from diabetes-susceptible mice. Fig S4 – Gga3 is a target of miR-335. Fig S5 – Molecular impact of miR-335-5p during 3T3-L1 differentiation.

Additional File 2: Tables S1-S9. Table S1: List of mature 150 miRNAs identified to be differnetially expressed in the diabetes-susceptible New Zealand Obese femlae mice. Table S2: Conservation of miRNA based on nucleotide sequence similarity in mouse and human. Table S3: miRNA-mRNA targets reported in 3 out of 5 databases and experimentally validated. Table S4: Putative protein targets for miRNAs. Both protein and miRNA are differentially expressed in the adipose tissue of the diabetes-susceptible mice. Table S5: Putative mRNA targets for miR-335-5p with fold changeand p value. Based on the known function of the gene, it was assigned to a particular categoryand CpGs annotated to miRNA genes that are also differnetially methylated. Table S6. 100 miRNAs in adipose tissue biopsies from 12 MZ twin pairs discordant for T2D matching with the differentially expressed miRNA in murine models. In bold P < 0.05, in red, the miRNA was newly identified, while the other have been presented in our previuos study. Table S7. Spearman statistics to assess correlation between the 100 miRNAs in adipose tissue biopsies from 12 MZ twin pairs discordant for T2D matching with the differentially expressed miRNA in murine models and blood glucose and HbA1c levels. Table S8. The 40 CpG sites in adipose tissue biopsies from 14 MZ twin pairs discordant for T2D corresponding to the 48 CpG sites from the mouse_MM285array annotated to the miRNA genes with differential expression in mouse models. In bold *P* < 0.05. The human data is based on our previous publication PMID: 24,812,430. Table S9. Spearman statistics to assess correlation between the 40 CpG sites in adipose tissue biopsies from 14 MZ twin pairs discordant for T2D corresponding to the 48 CpG sites from the mouse_MM285array and blood glucose and HbA1c levels. This analysis included both twins with and without T2D and is based on data from PMID: 24,812,430.

Additional File 3: Uncropped blot images.

## Data Availability

The majority of the conclusion made in this manuscript are based on data included in the Additional file 2 (Supplementary Tables S1-S9). Raw data from mice namely transcriptome, miRNome and methylome are available at the following GEO accession numbers: GSE310456, GSE310457 and GSE310390, respectively. Raw data of 3T3L1 gene expression are available at the following GEO accession number: GSE310455. Proteome results are deposited in the ProteomeXchange repository dataset identifiers PXD057291. Human data, including metabolic and molecular information, are already available in reference 20. Additional human data can be obtained from Charlotte Ling and Meriem Ouni upon reasonable request.

## Abbreviations

WAT: White adipose tissue
T2D: Type 2 diabetes
miRNA: MicroRNA
NZO: New Zealand Obese
TÜF: TÜbingen family study
HFD: High-fat diet
DP: Diabetes-prone
DR: Diabetes-resistant
WMCNA: Weighted miRNA co-expression network analysis
TF: Transcription factor
IR: Insulin resistance
*Mest*: Mesoderm-specific transcript protein
*Dlk1-Gtl2*: Delta like non-canonical notch ligand 1-Gene trap locus 2
CpG: 5′-Cytosine-phosphate-guanine-3′
ICR: Imprinting control region
