# Supplementary material for: Adipose tissue-derived microRNAs as epigenetic modulators of type 2 diabetes
Source: BMC Med. 2025 Dec 9;23:678. doi: 10.1186/s12916-025-04560-7 (PMC12690927; doi:10.1186/s12916-025-04560-7)
Supplement: Supplementary file 1 — Additional File 1: Methods. Expression proteomics analysis. Western blot analysis in 3T3-L1 cells. miRCURY assay-based validation for selected miRNAs & gene expression. Validation of GNA12 and GGA3 in SGBS cells. Demographic information of the human cohorts. Results. Chromosomal enrichment analysis for 150 differentially expressed miRNAs. Weighted miRNA co-expression network analysis- hub miRNAs & T2D. Figures S1-S5. Fig S1- Plasma insulin and free fatty acid levels. Fig S2 – miRCURY assay-based qPCR validation of selected miRNAs. Fig S3 – Snapshot of miRNA-mRNA-protein map of adipose tissue from diabetes-susceptible mice. Fig S4 – Gga3 is a target of miR-335. Fig S5 – Molecular impact of miR-335-5p during 3T3-L1 differentiation. [file 12916_2025_4560_MOESM1_ESM.docx]

**Additional file 1**

**Methods**

***Expression proteomics analysis***

For label free proteome analysis, frozen powdered gWAT tissue samples were lysed in 50 mM Tris–HCl, 150 mM NaCl and 1% sodium dodecyl sulfate (SDS) at pH 7.8 supplemented with 1 tablet cOmplete Mini and 1 tablet PhosSTOP (Roche, Basel, Switzerland) per 10 ml. Approximately 20 glass beads were added prior to disruption by sonication for 10 min (30 s on-off cycles at 4°C) using a Bioruptor Plus sonication device (Diagenode, Liège, Belgium). Protein concentrations were determined using the bicinchoninic acid assay (Pierce, Thermo Fisher Scientific, Rockford, USA). Afterward, cysteines were reduced by 30 min incubation at 56 °C with 10 mM dithiothreitol, and free sulfhydryl groups were alkylated with 30 mM iodoacetamide for 30 min at room temperature in the dark.

From this, 55 µg of sample were processed using S-trap Mini Column Digestion Protocol (PROTIFI, Farmingdale, NY, USA) according to the manufacturer’s instructions (1) with slight modifications. In brief, carbamidomethylated samples were diluted with 10% SDS to a final concentration of 5% SDS. Afterward, 6 μl of 12% phosphoric acid was added, followed by the addition of 420 μl of S-trap binding buffer (90% methanol, 100 mM triethylammonium bicarbonate (TEAB), pH 7.1). 200 μl of the acidified lysate/S-trap buffer mix was placed into the spin column and spun down in a bench-top centrifuge in a 2 ml tube at 4000g until all the solution had passed through. The flow through was discarded and the rest of the acidified lysate/S-trap buffer mix was loaded into the spin column and the procedure explained before was repeated.

Afterwards, 3 washing steps with 400 μl of S-trap binding buffer each were performed by centrifugation at 4,000 g. Then, sequencing grade modified trypsin (Promega, Madison, WI, U.S.A.) was added in an enzyme to a sample ratio of 1:20 (w/w) in 125 μl of 50 mM ammonium bicarbonate (ABC) containing 2 mM CaCl_2_. Spin columns were incubated for 1 h at 47 °C. After incubation, peptides were recovered by centrifugation prior addition of 80 μl of 50 mM ABC to the spin columns. Further peptide recovery was done by adding 80 μl 0.1% of trifluoroacetic acid (TFA) and 80 μl of 50% acetonitrile (for recovering hydrophobic peptides) to the spin columns followed by centrifugation. Finally, samples were dried under vacuum and resuspended in 0.1% TFA. Digestion quality control was performed via a monolithic column–HPLC (2). NanoLC–MS/MS analysis was done using a U3000 RSLCnano online-coupled (Thermo Scientific, Bremen, Germany) to a TIMS-TOF mass spectrometer (Bruker Daltonics, Bremen, Germany). Peptides were loaded onto the trap column (Acclaim PepMap100 C18; 75µm x 2 cm, Thermo Scientific, Bremen, Germany) in 0.1 % TFA at a flow rate of 10 µl/min. After 5 min, the pre-column was switched on‑line with the main column (IonOpticks, Aurora Ultimate CSI 25cm x 75µm, C18. Melbourne, Victoria, Australia) located in a separate column oven directly connected to the TIMS-TOF source at 50°C, peptides were separated using a 95 min binary gradient ranging from 3-42 % ACN in presence of 0.1 % FA at and a flow rate of 400 nL/min. MS was operated in Data-Independent acquisition- PASEF mode. The setting for MS1 were as follows, scan from 100 -1700 m/z, positive polarity, scan mode DIA-PASEF. TIMS setting mobility 0.6 to 1.51 V.s/cm^2^ with a ramp time 100 ms and 100% duty cycle. Windows for fragmentation were set as shown in the Figure.


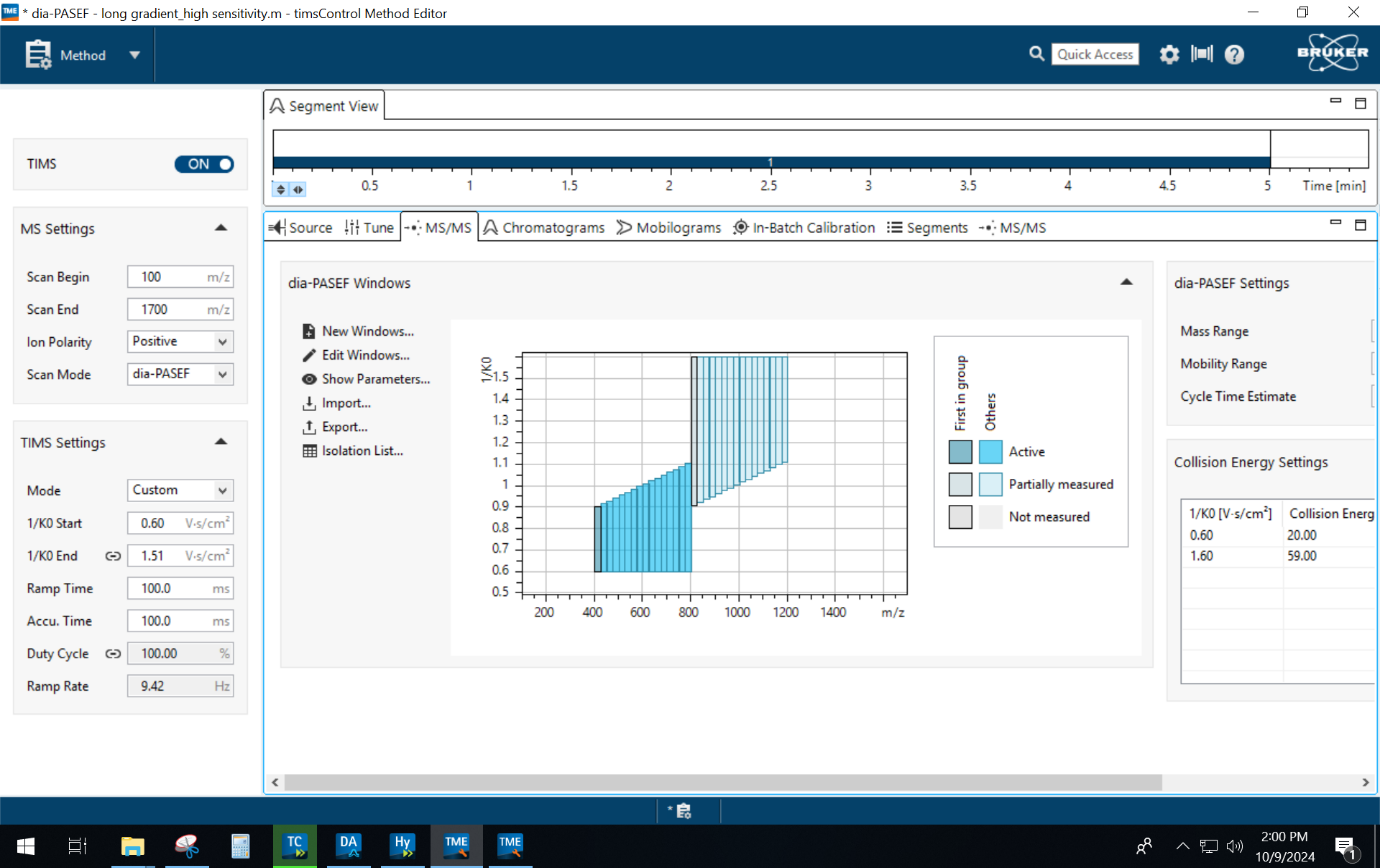
Raw data was analyzed using Spectronaut Pulsar (Biognosys), using a mouse database (swissPROT _tremBl) and the following parameters for direct-DIA (Deep) analysis, enzyme was set to trypsin/P, missed cleavages 2, fixed modification Carbamidomethyl (C +57 Da), and variable modification Acetyl (Protein N-term 42 Da), Oxidation (M +16 Da) A cross-run normalization approach was used with normalization strategy set on automatic. Results files were exported, based on PG protein quantification log 2 ratio as well as p-values were calculated between diabetes-resistant versus diabetes-prone samples. Raw data and Proteome Discoverer search results are deposited in the ProteomeXchange repository dataset identifiers PXD057291. Reviewer access details - Log in to the PRIDE website using the following details: Project accession- PXD057291 and Token- oXHPYE9FXT7E. Alternatively, reviewer can access the dataset by logging in to the PRIDE website using the following account details: Username- reviewer_pxd057291@ebi.ac.uk and Password- AMajtxhzVjvq.

***Western blot analysis in 3T3-L1 cells***

Protein lysates were prepared from 3T3-L1 cells as described in section 2.9. Western blot analysis was performed using 15 mg protein per sample with primary antibody recognized Akt (Cell Signaling Technologies, #2920), pAkt_S473_ (Cell Signaling Technologies, #9271), GLUT4 (self-made) or α-Tubulin (Sigma-Aldrich, #T6074) and secondary antibody (LiCOR, P/N 926-68070, P/N 926-68071, P/N 926-32212).

***miRCURY assay-based validation for selected miRNAs & gene expression***

The table below contains the catalog information and mature miRNA sequence of the assayed miRNAs and forward and reverse sequence of the genes.

| **miRNA** | **Catalog number** | **mature miRNA sequence** |
| --- | --- | --- |
| hsa-miR-142-5p | YP00204722 | CAUAAAGUAGAAAGCACUACU |
| hsa-miR-146b-5p | YP02119310 | UGAGAACUGAAUUCCAUAGGCUG |
| hsa-miR-335-5p | YP02119293 | UCAAGAGCAAUAACGAAAAAUGU |
| mmu-miR-342-5p | YP00205185 | AGGGGUGCUAUCUGUGAUUGAG |
| **Gene** | **Forward primer** | **Reverse primer** |
| *Gna12* | AAGTCCACCTTCCTCAAGCA | CCATCAGAAACATCCCGTGC |
| *Hspa13* | TGCCTGCAGAATTCGACCTA | GAGTTCCTCCACCCAAGTCT |
| *Gga3* | CTACCAATCCTTCCAACCGC | TGAAGTCTCCTGCCACAGTT |
| *Ppil2* | CGAGAGAGACGCTACAGGAG | ACCATGGCAGTAGAGGTGAA |

***Validation of GNA12 and GGA3 in SGBS cells***

Human Simpson-Golabi-Behmel syndrome (SGBS) cells were cultured as described (3). One day after seeding, cells were transfected with 20nM hsa-miR-335-5p mimic (C-300708-05-0005, Horizon) or 20 nM non-targeting (NT) control (CN-001000-01-20, Horizon) by using lipofectamine 2000 according to the manufacturer's protocol (11668019, Invitrogen). After 48 h of incubation, cells were harvested for RNA isolation (day 0).

Total RNA was purified with Quick-RNA Miniprep Kit (R1055, Zymo), reverse transcribed with SuperScript II Reverse Transcriptase (18064071, Invitrogen) and quantified with iTaq Universal SYBR Green Supermix (1725124, BioRad) using the following primers: EEF2-fwd: 5’-CAT TGC CGA GCG CAT CAA G-3’; EEF2-rev: 5’-CTC GCC GTA GGT GGA GAT GA-3#; GGA3-fwd: 5’-GAT AGG ACG CTG ATC CCC TCT-3’; GGA3-rev: 5’-TCA CTG AGC AGT CTC ACG TTG-3’; GNA12-fwd: 5’-CCG CGA GTT CGA CCA GAA G-3’; GNA12-rev: 5’-TGA TGC CAG AAT CCC TCC AGA-3’.

To quantify the miR-335-5p level, the purified RNA was reverse transcribed using the miRCURY LNA RT Kit (339340, Qiagen) and measured with the miRCURY LNA SYBR Green Kit (339347, Qiagen) by using the following primers: hsa-SNORD44 (YP00203902, Qiagen) and hsa-miR-335-5p (YP02119293, Qiagen).

***Demographic information of the human cohorts***

The table below contains the demographic information for the monozygotic twin pairs and the females at high risk of developing T2D from German cross-sectional TÜbingen Family (TÜF) study.

|  | **Monozygotic Twin pairs discordant for T2D** | **TÜF study** |
| --- | --- | --- |
| N (singletons, twin pairs) | 28 (14) | 99 |
| Age (years), mean ± SD | 68.1 ± 8.1 | 50.3 ± 11.6 |
| Female, % | 10 (35.7%) | 100% |
| Smoking, % (N =18) | Yes 3 (10.7%), No 15 (53.6%) | Not available |
| BMI (kg/m^2^), mean ± SD | 30.7 ± 7.0 | 31.4 ± 5.3 |

**Results**

***Chromosomal enrichment analysis for 150 differentially expressed miRNAs***

The table below shows the p value for the chromosomal enrichment analysis for the data presented in Figure 4A using the Fischer’s exact test.

| **Chromosome** | **No. of differentially expressed miRNAs** | **p-value** |
| --- | --- | --- |
| chr1 | 13 | 0.04 |
| chr2 | 13 | 0.1117 |
| chr3 | 1 | 0.0094 |
| chr4 | 6 | 0.4608 |
| chr5 | 2 | 0.0213 |
| chr6 | 4 | 0.3574 |
| chr7 | 9 | 0.3458 |
| chr8 | 1 | 0.0058 |
| chr9 | 10 | 0.1128 |
| chr10 | 3 | 0.0928 |
| chr11 | 17 | 0.0316 |
| chr12 | 25 | 0.0001 |
| chr13 | 8 | 0.0629 |
| chr14 | 5 | 0.567 |
| chr15 | 4 | 0.2464 |
| chr16 | 6 | 0.25 |
| chr17 | 1 | 0.0212 |
| chr18 | 6 | 0.2286 |
| chr19 | 3 | 0.2964 |
| chrX | 13 | 0.3297 |

***Weighted miRNA co-expression network analysis- hub miRNAs & T2D***

The table below contains the literature search results for the miRNAs identified using WMCNA as hub miRNAs for all the 13 modules.

The hub miRNAs from the WMCNA analysis were screened for their known functional relevance in the pathophysiology of type 2 diabetes (T2D).

| **miRNA_ID** | **References** | **Established functional role in the**  **pathogenesis of T2D** |
| --- | --- | --- |
| mmu-miR-222-5p | https://doi.org/10.1016/j.omtn.2022.02.005 | YES |
| mmu-miR-221-3p | https://doi.org/10.1016/j.omtn.2022.02.005 | YES |
| mmu-miR-148a-3p | https://doi.org/10.1007/s00592-020-01569-7; https://doi.org/10.1186/s12902-022-01120-5 | YES |
| mmu-miR-431-5p | https://doi.org/10.1016/j.ajpath.2019.11.007; https://doi.org/10.1016/j.xcrm.2024.101591 | NO |
| mmu-miR-381-3p | https://doi.org/10.1016/j.bcp.2021.114473; https://doi.org/10.1002/jgm.3274 | YES |
| mmu-miR-222-3p | https://doi.org/10.1016/j.ebiom.2016.12.002; https://doi.org/10.3389/fendo.2021.750261; https://doi.org/10.1186/s12905-022-01912-w | YES |
| mmu-miR-204-5p | https://doi.org/10.1016/j.gene.2018.05.036 | YES |
| mmu-miR-21a-3p | https://doi.org/10.1016/j.gene.2023.147212 | YES |
| mmu-miR-203-3p | https://doi.org/10.1016/j.isci.2022.104708 | YES |
| mmu-miR-142a-3p | https://doi.org/10.1016/j.mce.2020.111028 | YES |
| mmu-miR-23b-5p | https://doi.org/10.1016/j.molmet.2017.04.006 | YES |
| mmu-miR-126a-3p | https://doi.org/10.1016/j.molmet.2021.101306; https://doi.org/10.3390/diagnostics11060949; https://doi.org/10.1038/srep36207 | YES |
| mmu-miR-574-5p | https://doi.org/10.1016/j.omtn.2021.08.031; https://doi.org/10.1080/09513590.2021.1908990 | YES |
| mmu-miR-300-3p | https://doi.org/10.1016/j.omtn.2023.03.016 | NO |
| mmu-miR-141-3p | https://doi.org/10.1016/j.omtn.2024.102163 | YES |
| mmu-miR-34b-3p | https://doi.org/10.1016/j.tranon.2024.102063; https://doi.org/10.4093/dmj.2020.0019; https://doi.org/10.12659%2FMSM.917128 | YES |
| mmu-miR-149-5p | https://doi.org/10.1016/j.yexmp.2019.104279; https://doi.org/10.3389/fonc.2021.743077; https://doi.org/10.1159/000480330 | YES |
| mmu-miR-30e-5p | https://doi.org/10.1038/s41366-018-0114-1; https://doi.org/10.1007/s00125-014-3434-2 | YES |
| mmu-miR-34c-3p | https://doi.org/10.1038/s41392-020-0133-y; https://doi.org/10.18632%2Foncotarget.21883 | YES |
| mmu-miR-199a-5p | https://doi.org/10.1038/s41419-018-0439-7;  https://doi.org/10.3390%2Fcells10123342 | YES |
| mmu-miR-214-5p | https://doi.org/10.1038/s41419-018-0752-1 | NO |
| mmu-miR-1199-5p | https://doi.org/10.1038/s41467-017-01197-w | NO |
| mmu-miR-181b-5p | https://doi.org/10.1038/s41598-017-13875-2; https://doi.org/10.1161/CIRCRESAHA.115.308166 | NO |
| mmu-let-7c-1-3p | https://doi.org/10.1073/pnas.1118922109 | YES |
| mmu-miR-184-3p | https://doi.org/10.1096/fj.201701100R; https://doi.org/10.1038/s41420-022-01142-x | YES |
| mmu-miR-212-3p | https://doi.org/10.1111/jcmm.12733; https://doi.org/10.3892/ijmm.2023.5219 | YES |
| mmu-miR-146b-5p | https://doi.org/10.1371/journal.pone.0032794; https://doi.org/10.3390/antiox10010101 | YES |
| mmu-miR-500-3p | https://doi.org/10.18632/oncotarget.23651 | NO |
| mmu-miR-29a-3p | https://doi.org/10.2337/db13-1015; https://doi.org/10.1016/j.celrep.2020.108576 | YES |
| mmu-miR-29b-3p | https://doi.org/10.2337/db13-1015; https://doi.org/10.1016/j.celrep.2020.108576; https://doi.org/10.1111%2Fjcmm.14030 | YES |
| mmu-miR-708-3p | https://doi.org/10.2337/db16-1569; https://doi.org/10.3390/ijms20246358 | YES |
| mmu-let-7f-5p | https://doi.org/10.2337/db20-0324 | YES |
| mmu-miR-342-3p | https://doi.org/10.2337/db20-0324; https://doi.org/10.3389/fendo.2021.727915; https://doi.org/10.1159/000374032 | YES |
| mmu-miR-455-5p | https://doi.org/10.2337/db21-0134; https://doi.org/10.15252/embr.201540837 | YES |
| mmu-miR-455-3p | https://doi.org/10.2337/db21-0134; https://doi.org/10.15252/embr.201540837 | YES |
| mmu-miR-99b-5p | https://doi.org/10.2337/db21-306-OR; https://doi.org/10.3389/fendo.2019.00840 | YES |
| mmu-miR-26a-5p | https://doi.org/10.26444/jpccr/128009; https://doi.org/10.2147/DMSO.S335088; https://doi.org/10.1038/s41433-021-01393-5 | YES |
| mmu-miR-376b-3p | https://doi.org/10.33696%2Fdiabetes.1.003 | YES |
| mmu-miR-135a-5p | https://doi.org/10.3389/fendo.2023.1035029; https://doi.org/10.3892/ijmm.2020.4647 | YES |
| mmu-miR-671-5p | https://doi.org/10.3389/fmolb.2022.1077968 | NO |
| mmu-miR-18a-3p | https://doi.org/10.3390/diagnostics13142443; https://doi.org/10.18632/aging.202319 | YES |
| mmu-miR-186-5p | https://doi.org/10.3390/ijms22147712 | NO |
| mmu-miR-10b-5p | https://doi.org/10.3390/ijms251810147 | YES |
| mmu-miR-511-5p | https://doi.org/10.3390/jcm11030805 | NO |
| mmu-miR-328-3p | https://doi.org/10.4093/dmj.2021.0216 | NO |
| mmu-miR-3057-5p | NA | NO |
| novel_mir59 | NA | NO |
| novel_mir66 | NA | NO |
| mmu-miR-700-5p | NA | NO |
| novel_mir29 | NA | NO |

**Figures**

**
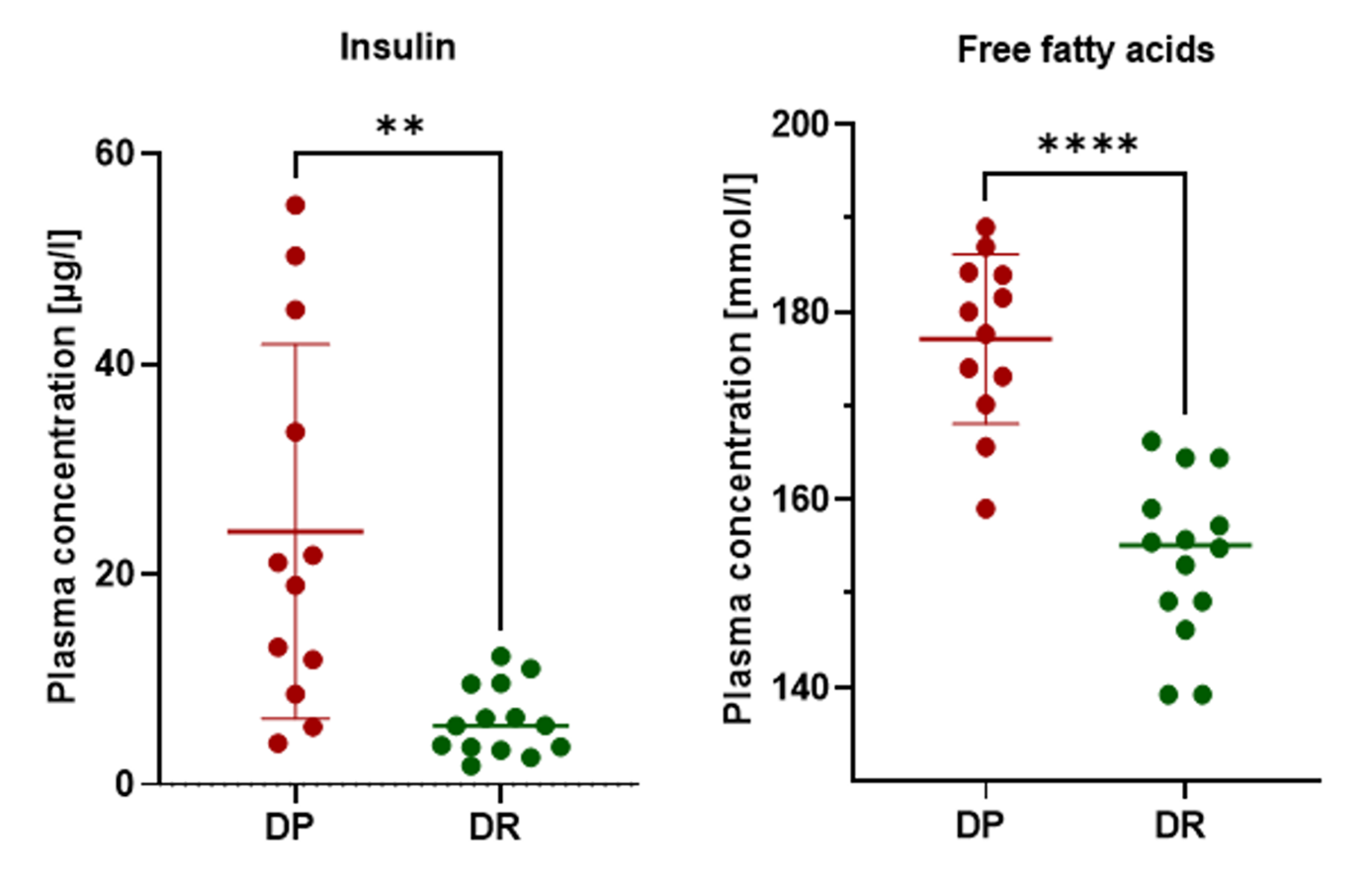
**

**Fig. S1: Plasma insulin and free fatty acid levels.** Plasma insulin concentration in µg/l was measured with high-sensitivity ELISA and free fatty acids in mmol/l. Data represented as mean ± SD for DP (red) and DR (green) animals; Unpaired t-test with Welch’s correction; **p<0.005, ****p<0.0001.

**
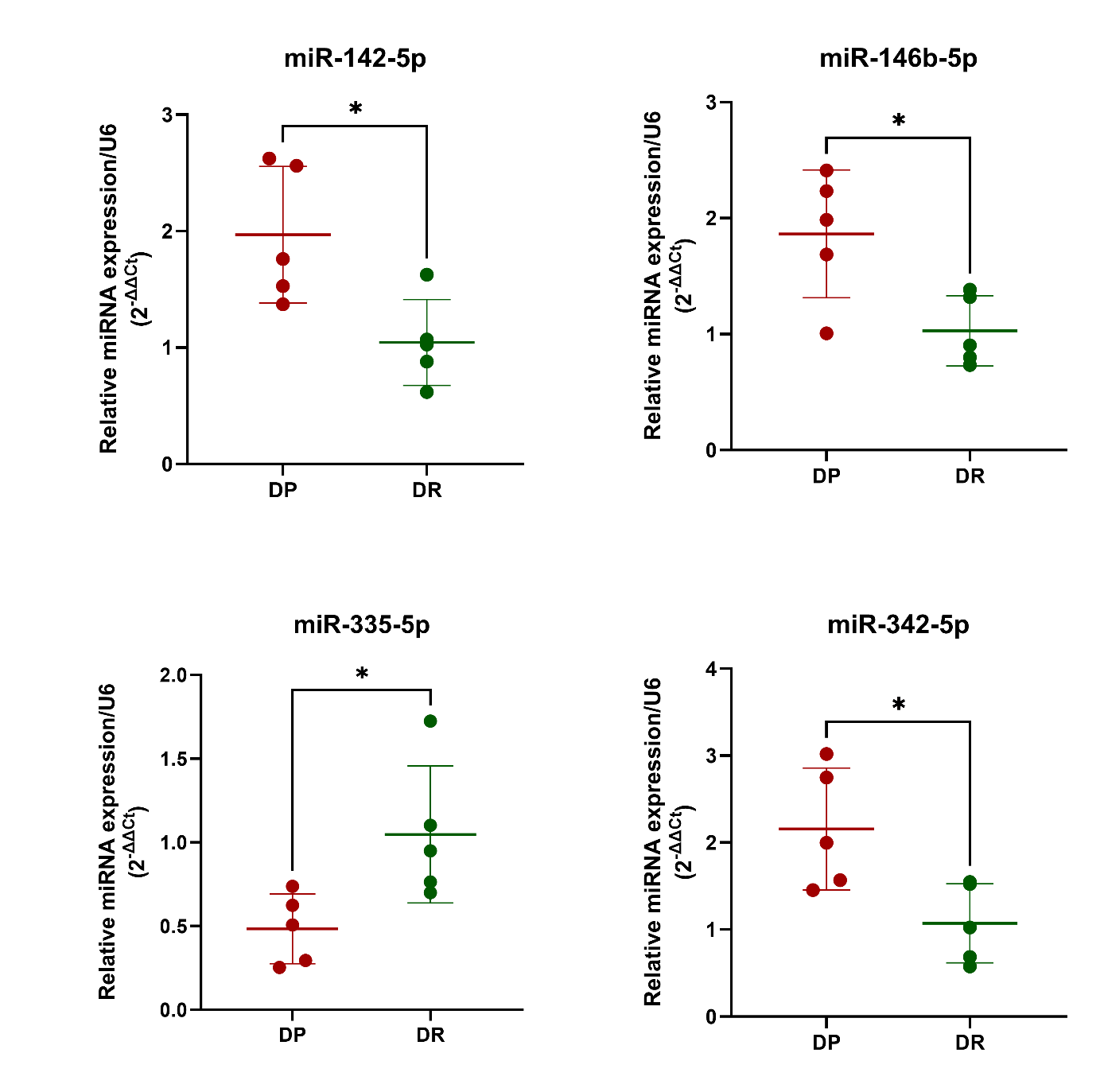
**

**Fig. S2: miRCURY assay-based qPCR validation of selected miRNAs.** A subset of mice from the DP and DR group were included for validation and *U6* was used as the endogeneous control. Data represented as mean ± SD; n = 6/group; Unpaired t-test with Welch’s correction; *p < 0.05.

**
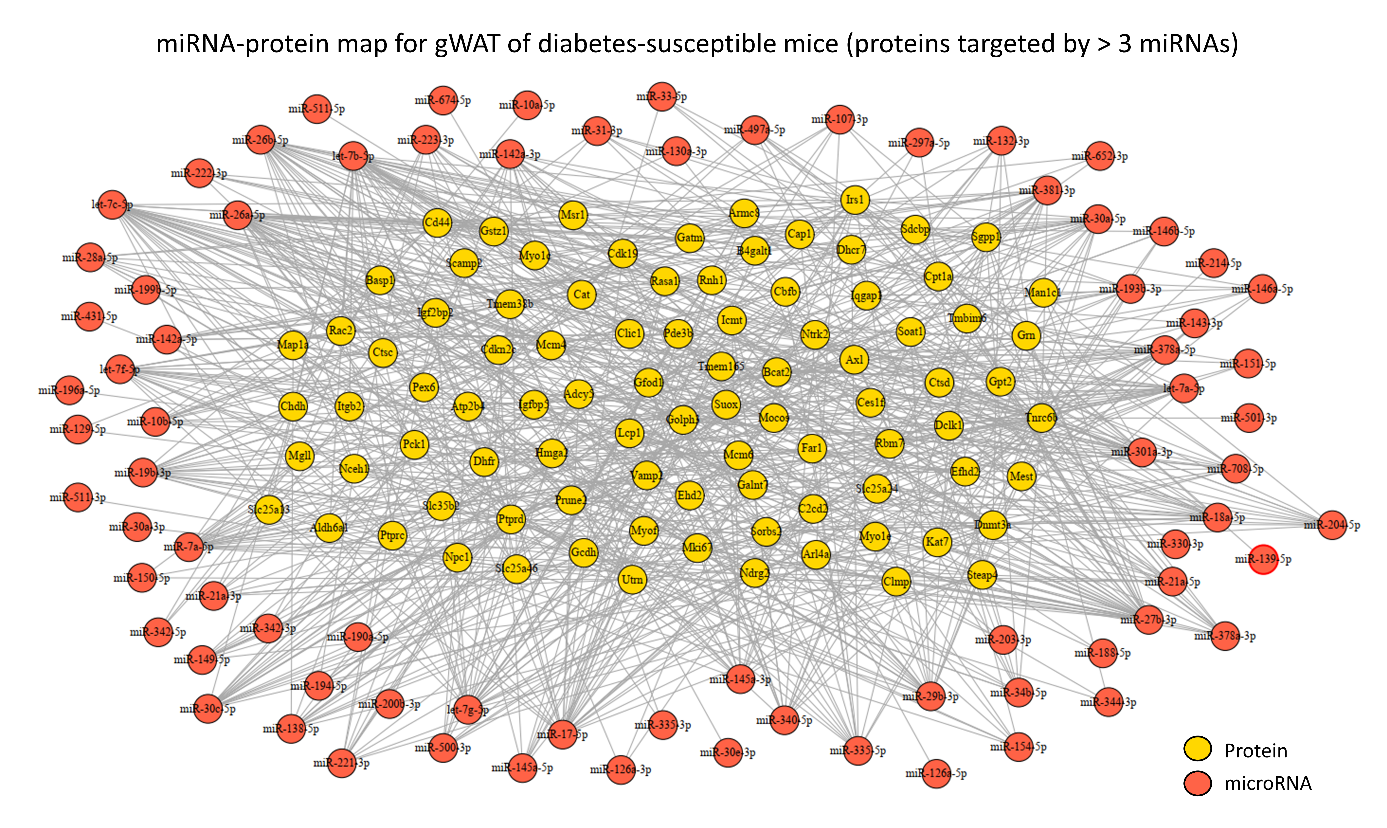
**

**Fig. S3: Snapshot of miRNA-mRNA-protein map of adipose tissue from diabetes-susceptible mice.** MiRNA-protein network based on differentially expressed miRNAs and proteins that are also differentially expressed in gWAT of DP mice. Circles represent miRNA or protein and lines represent putative regulation for proteins putatively targeted by > 3 miRNAs.

**
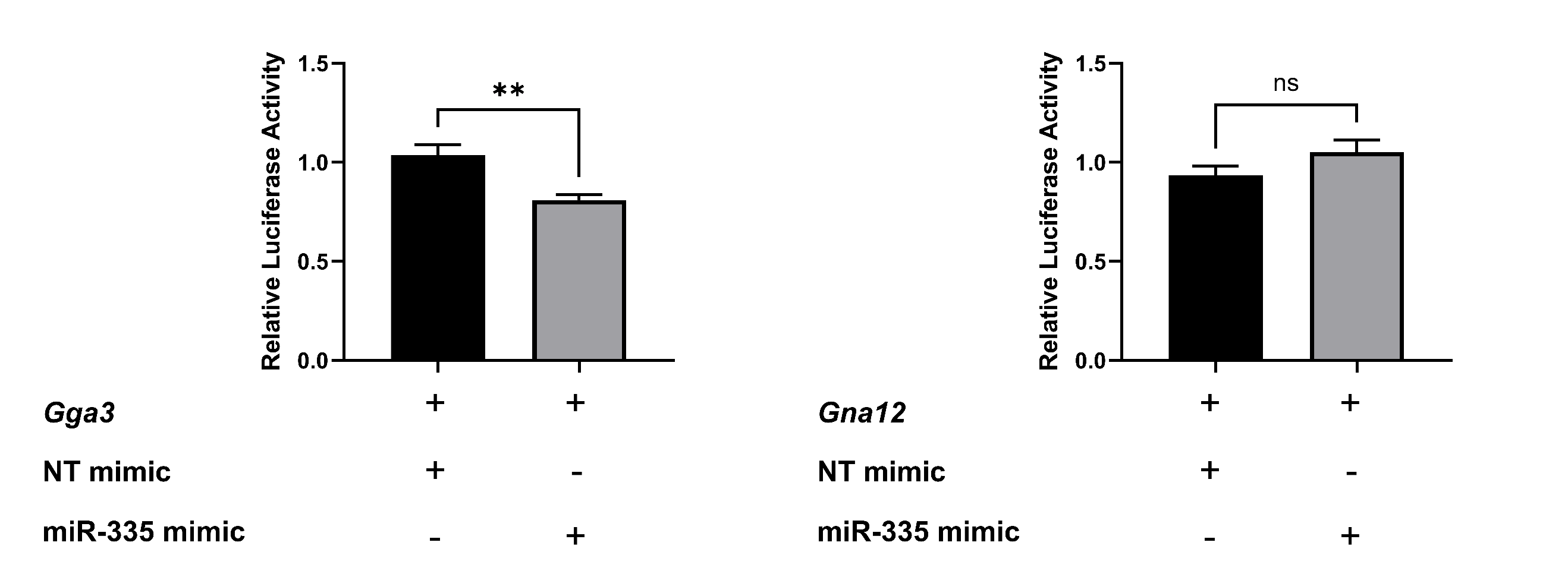
**

**Fig. S4: Gga3 is a target of miR-335.** Levels of luciferase activity in HeLa cells overexpressing miR-335-5p mimic and transfected with the Gga3 vector. Data are indicated as ratios with respect nontargeting miRNA. All luciferase activity data are presented as mean ± SEM. values from three independent experiments, each performed in triplicate (p < 0.05 vs NT mimic, two-tailed Mann–Whitney U test).

**
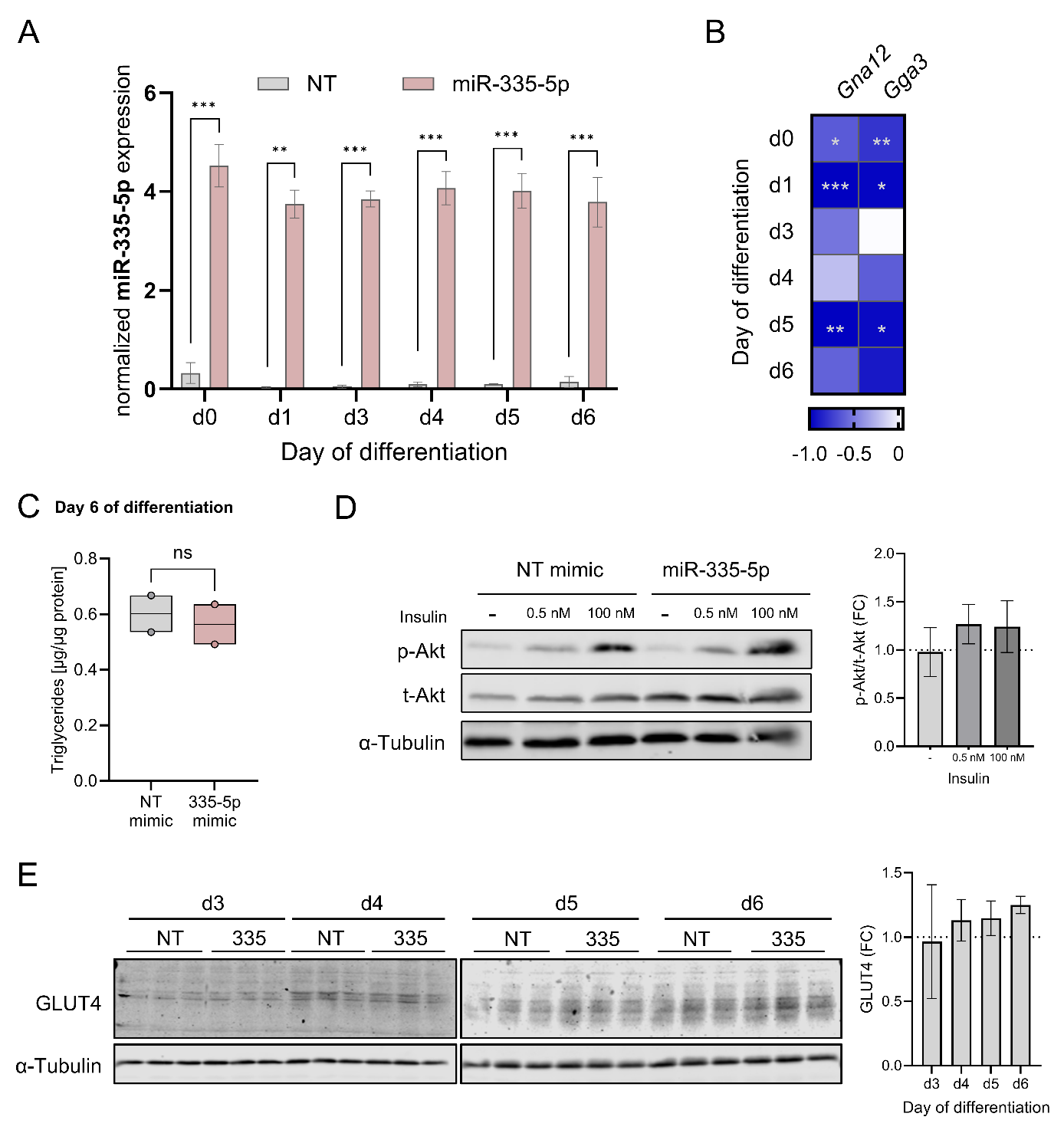
**

**Fig. S5: Molecular impact of miR-335-5p during 3T3-L1 differentiation.** (A) Expression profile of miR-335-5p during 3T3-L1 differentiation. (B) Heatmap showing Pearson correlation statistics, with color intensity representing the correlation coefficient (r) and asterisks indicating statistical significance (p-values). (C) Triglyceride accumulation in 3T3-L1 cells at day 6 of differentiation following transfection with NT or miR-335-5p. (D) Western blots of insulin-stimulated (0 nM, 0.5 nM or 100 nM for 20 min) mature adipocytes transfected with NT mimic or miR-335-5p mimic, respectively (left) and corresponding quantification plots showing the fold change (right). (E) Western blots of GLUT4 expression during differentiation (day 3, day 4, day 5 and day 6) of 3T3-L1 cells transfected either with NT mimic or miR-335-5p mimic (left) and quantification plots showing the fold change (right). Unpaired t-test (A); unpaired t-test with Welch’s correction (C); Data showing the mean ± SEM for two to six independent experiments (A, D and E); ns - not significant, *p < 0.05, **p < 0.01, ***p < 0.001.

**References**

1. Ludwig KR, Schroll MM, Hummon AB. Comparison of In-Solution, FASP, and S-Trap Based Digestion Methods for Bottom-Up Proteomic Studies. J Proteome Res. 2018 Jul 6;17(7):2480–90.

2. Burkhart JM, Schumbrutzki C, Wortelkamp S, Sickmann A, Zahedi RP. Systematic and quantitative comparison of digest efficiency and specificity reveals the impact of trypsin quality on MS-based proteomics. J Proteomics. 2012 Feb;75(4):1454–62.

3. Wabitsch M, Brenner RE, Melzner I, Braun M, Möller P, Heinze E, et al. Characterization of a human preadipocyte cell strain with high capacity for adipose differentiation. Int J Obes Relat Metab Disord. 2001;25(1):8–15.
