# Supplementary figures and images for: Adipose tissue-derived microRNAs as epigenetic modulators of type 2 diabetes
Source: BMC Med. 2025 Dec 9;23:678. doi: 10.1186/s12916-025-04560-7 (PMC12690927; doi:10.1186/s12916-025-04560-7)

Additional File 3: Uncropped blot images.

Fig. S5D

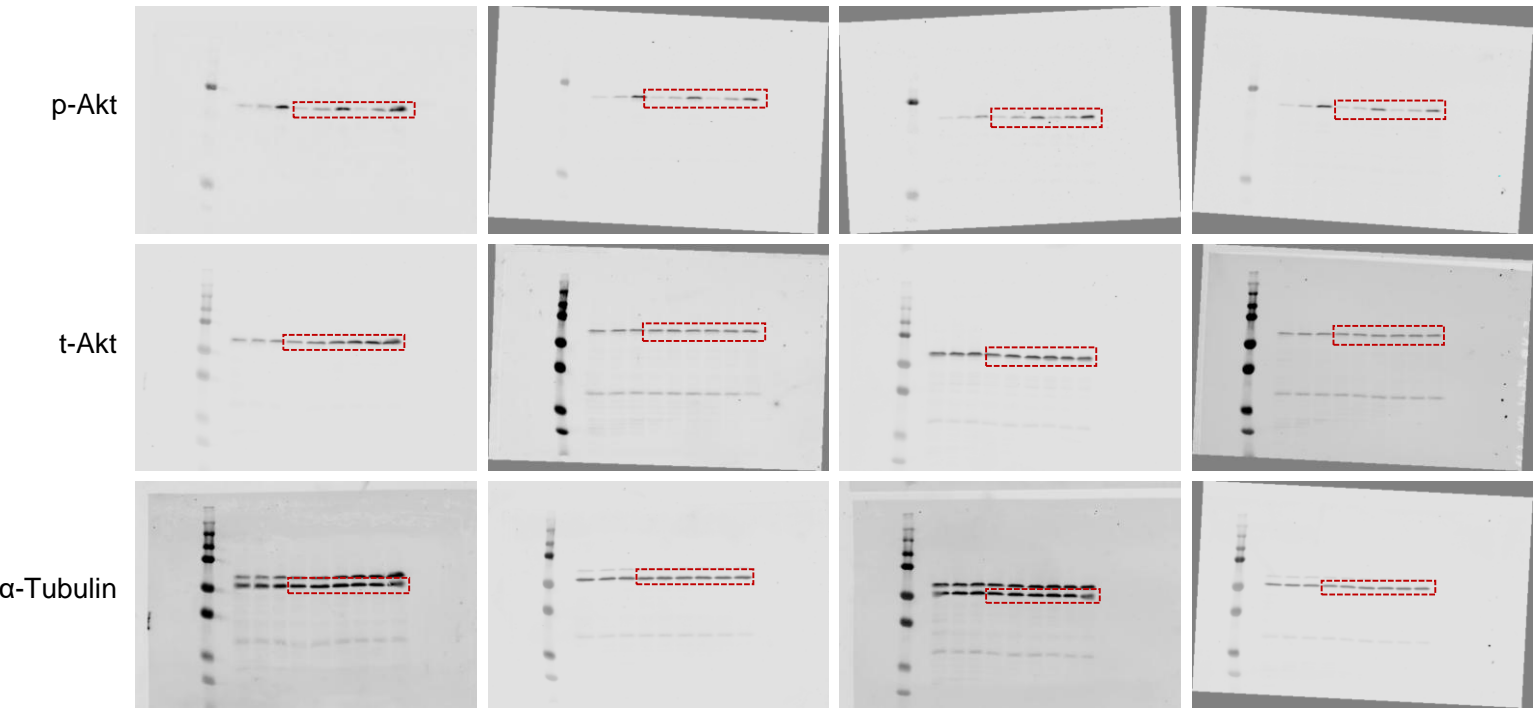

Fig. S5E

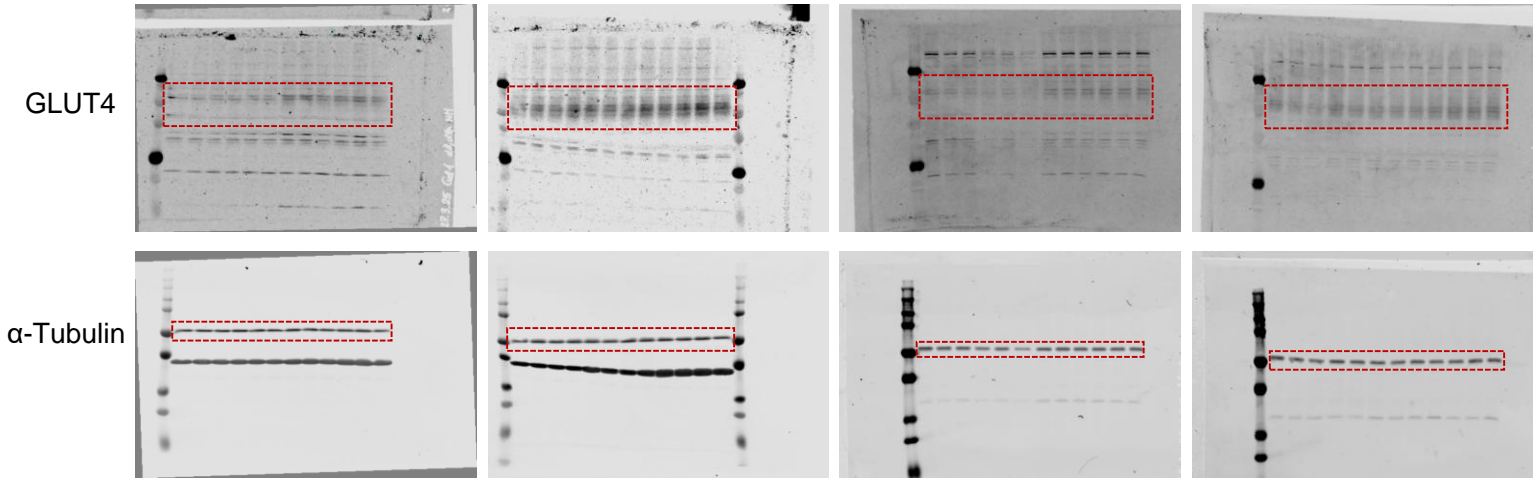

Supplement: Supplementary file 3 — Additional File 3: Uncropped blot images. [file 12916_2025_4560_MOESM3_ESM.pdf]
